# Acceptability of School Menus: A Systematic Review of Assessment Methods

**DOI:** 10.3390/ijerph20032242

**Published:** 2023-01-27

**Authors:** Síntia Almeida Santana, Sueny Andrade Batista, Dayanne da Costa Maynard, Verônica Cortez Ginani, Renata Puppin Zandonadi, Raquel Braz Assunção Botelho

**Affiliations:** Department of Nutrition, University of Brasília, Brasília 70910-900, Brazil

**Keywords:** food consumption, students, school feeding, acceptability

## Abstract

The school meal promotes healthy eating habits through nutritionally adequate preparations. Consequently, it prevents obesity and malnutrition, favoring learning. This systematic review aimed at investigating the methods for evaluating the acceptance of school menus offered by school feeding programs (SFPs) around the world. Specific search strategies were conducted on PubMed, Lilacs, Web of Science, Scopus, Embase, Google Scholar, and ProQuest Global. The methodological quality of the included studies was assessed using the Meta-Analysis Statistical Assessment and Review Instrument. A total of 89 studies were included. The countries with the highest number of studies were Brazil (*n* = 42), South Korea (*n* = 13), and the United States (*n* = 12). The most used methods (69.66%) were numerical scales, with a higher prevalence for the 5-point scale (50.56%). Other methods included questionnaires and/or interviews with objective and/or subjective questions (44.94%); and mathematical formulas and/or visual estimates evaluating the consumption of food and leftovers (40.45%). The prevalent use of the 5-point hedonic scale may be due to its low cost, simplicity, ease of elaboration, application speed, and student understanding. Mathematical formulas and/or visual estimation were used by about 40% of the studies, but it is difficult to compare studies.

## 1. Introduction

School feeding programs (SFPs) are social policies within the scope of food and nutrition security (FNS) generally aimed at children and adolescents in situations of food insecurity and living in areas of low socioeconomic status [1], corroborating the importance of these programs to guarantee the food security of these individuals. By early 2020, 388 million had received school meals daily in at least 161 countries of all income levels [2].

However, The State of Food Security and Nutrition in the World 2021 report, a global assessment of food insecurity and malnutrition in 2020, estimated that 2.3 billion people, nearly two-thirds of the global population, lack access to adequate food. Furthermore, between 720 and 811 million faced hunger, and the prevalence of malnutrition reached approximately 9.9% of the population worldwide [3].

These programs provide, through school meals, a social safety net and lead to improved educational and nutrition outcomes [4]. This network includes immediate responses to economic shocks and long-term social protection. They benefit educational performance by increasing school enrollment and attendance, reducing absenteeism, and contributing to learning and cognition. In addition, they cooperate to alleviate hunger and provide nutrients to fight malnutrition. According to [5], these programs offer nutritionally balanced meals that contribute to developing healthy eating behaviors among students.

However, several studies report that the acceptance of the offered school menus/meals by these programs may not correspond to the expectations of the planning stage and, thus, not reach the objectives of being effectively consumed and appreciated [6,7,8,9,10,11,12,13,14,15].

Given this scenario, the numerous factors involved in the acceptance of school meals are well-founded in the literature. Among them, there are sensory characteristics, such as taste [16,17,18,19] and appearance, including color, size, and form [20,21], food preferences and neophobias [22,23,24], and the consumption of competitive foods (sold in school cafeterias, purchased outside of school, and brought from home) [25,26,27]. Additionally, the physical and cultural environment and sociodemographic characteristics such as sex, race, education level, and family income influence acceptance [28,29].

The listed factors may increase school food waste [30,31,32,33]. The amount of food waste, among other factors, is related to students’ perception and acceptance of school meals and eating habits in the home environment [34,35,36].

According to the Food Waste Index report (2021) by the United Nations Environment Program (UNEP), food waste in households, retail establishments, and the food service industry totaled 931 million tonnes per year. The global average of 74 kg per capita of food wasted yearly is similar across low, middle, and high-income countries [37]. Faced with this problem, Target 12.3 of the Sustainable Development Goals (SDGs) by the United Nations Organization (UN) aims to halve per capita food waste worldwide, at retail and consumer levels, and reduce food loss by 2030 [38].

The literature on the subject is broad and diverse, and there is no consensus on which methodologies should be used to effectively measure school menus’ acceptance. Because of this, the importance of adequate instruments and methods for collecting acceptance data is reinforced so that it is possible to verify the effectiveness of these programs. For advances in the field, synthesizing the literature is an important step, and there is evidence that there is no study in the scientific literature that verifies the existence of methods and assessment instruments across countries. Therefore, the objective of the present systematic review was to investigate the methods for evaluating the acceptance of school menus offered by SFPs in different countries.

## 2. Materials and Methods

This systematic review was prepared according to the Preferred Reporting Items for Systematic Reviews and Meta-Analyses (PRISMA), and its checklist (PRISMA) was registered in PROSPERO (CRD42022321616). The protocol was performed according to the following steps.

### 2.1. Inclusion and Exclusion Criteria

The inclusion criteria were studies that evaluated the acceptance of school menus offered by SFPs up to high schools worldwide, with no date and language limits. The exclusion criteria were: (i) comments, reviews, letters, abstracts, conferences, undergraduate papers, clinical and review studies, case reports, and books, (ii) studies that do not focus on the evaluation of acceptance of school menus from SFPs around the world, (iii) studies that analyzed the acceptance of preparations that have not yet been included in the menus of the SFPs, (iv) studies in private schools, unrelated to government-subsidized SFPs, (v) preliminary studies, and (vi) studies that evaluated the acceptance of menus offered in universities (Table S1 in Supplementary Materials). No filter on publication date was used since we aimed to search for any acceptability of school menus assessment methods previously published.

### 2.2. Information Source

Detailed individual search strategies were developed for each database: Pubmed, Lilacs, Web of Science, Scopus, and Embase. A search for gray literature was performed on Google Scholar and for dissertations and theses in ProQuest Global. In addition, the reference lists of selected articles were examined to read the full text of possible relevant studies, as these could have been missed during the electronic search in databases. The last search in all databases was carried out on 11 January 2023.

### 2.3. Search Strategy

The appropriate combinations of truncation and keywords were selected and adapted for the search in each database (Table S2 in Supplementary Materials). Rayyan software (Qatar Computing Research Institute-QCRI) was used to assist in selecting and excluding duplicate articles, and all references were managed by Mendeley desktop software.

### 2.4. Study Selection

The process of screening the studies was carried out in two phases. In phase 1, two researchers (SAS, SAB) independently reviewed the titles and abstracts of all references identified in the databases. These excluded articles that did not meet the eligibility criteria. In phase 2, the full texts of the selected articles were read in full by the same reviewers (SAS, SAB), and only those that met the inclusion criteria were included. In cases of divergence, for both phases, there was discussion until a consensus was reached between the two reviewers. Otherwise, a third reviewer (DdCM) made the final decision. The final selection was based on the full text. SAS critically evaluated the reference list of selected studies. Additional studies were added by the experts (RBAB, RPZ).

### 2.5. Data Collection Process

Two reviewers (SAS, SAB) independently collected the following characteristics from the selected studies: authors and year of publication, country of research, the objective of the study, methods and/or strategies/protocols for sensory evaluation and acceptance of school menus offered by SFPs in the world, and main results referring to the identified methods. Calibration exercises were performed before starting the review to ensure consistency across reviewers. Disagreements were resolved by discussion, and the third reviewer (DdCM) judged the disagreements. These data were synthesized by three reviewers (SAS, SAB, and DdCM) using a standardized table containing the following information: references, authors, year, country, objectives, schools (quantity), teaching stage (according to the teaching stages of each country), participants (students, parents, nutritionists, employees), acceptance methods performed, evaluated attributes, and main results referring to the identified methods.

### 2.6. Risk of Individual Bias in the Included Studies

The quality criteria were synthesized using a statistical review assessment instrument (MASTARI) and the Joanna Briggs Institute protocol to assess the risk of bias in the studies. The instrument for assessing the risk of bias included seven questions:

1. Were the methods of evaluation of acceptance of menus characterized?
2. Were the evaluated menus and/or preparations specified?
3. Was the evaluation carried out in schools participating in school feeding programs?
4. Was the study design adequate?
5. Was the sample of participants selected for the analysis representative and randomly determined?
6. Was the statistical analysis adequate for the objective of the study?
7. Did the results answer the main question?

After analysis, the risk of bias was categorized according to the percentage of “yes” scores: “High” for up to 49%, “Moderate” for between 50 and 69%, and “Low” for more than 70% (Table S3 in Supplementary Materials).

## 3. Results

**Figure 1:**
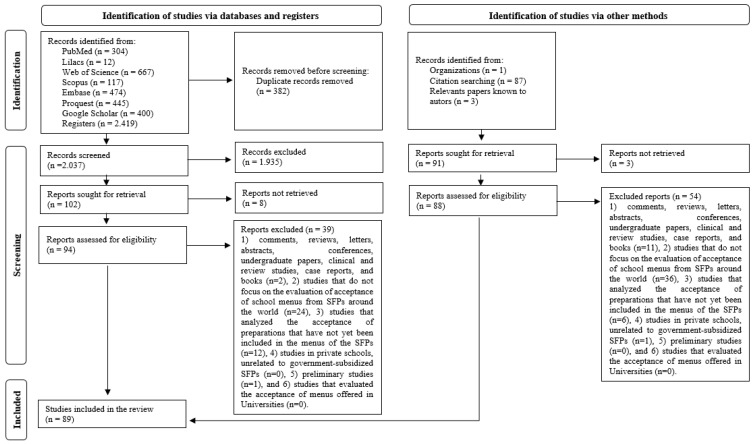
Flowchart of included/excluded studies and search conducted on school menus assessment methods. Adapted from the PRISMA protocol (2020).

Of the 2419 studies found, and after excluding 382 duplicates, 2037 were selected through their abstracts. Of these, 1935 were excluded for not meeting the eligibility criteria. Thereby, 102 studies were selected for a full reading. However, 8 studies were not found, totaling 94 eligible studies. After reading, 39 were excluded, and 55 studies were included. Other studies were identified through other methods, citation searching (*n* = 87), relevant papers known to authors (*n* = 3), and organizations (*n* = 1), totaling 91 studies thoroughly read. However, 3 were not found, resulting in 88 eligible studies, of which 54 were excluded, and 34 were included. In the end, the 55 previously included were added to these 34, resulting in a total of 89 articles in the systematic review (Figure 1).

### 3.1. Studies Characteristics

The selected studies were conducted in the following countries: Brazil (*n* = 42), South Korea (*n* = 13), the United States (*n* = 12), Italy (*n* = 8), Colombia (*n* = 2), India (*n* = 2), Chile (*n* = 3), Ghana (*n* = 1), Philippines (*n* = 1), Spain (*n* = 1), Paraguay (*n* = 1), Finland (*n* = 1), and Georgia (*n* = 1). The date range for the included studies was between 1977 and 2021 (Table 1).

The 89 studies included in this review were extracted from articles published in scientific journals (85.39%; *n* = 76), master’s dissertations (6.74%; *n* = 6), doctoral theses (5.62%; *n* = 5), and governmental organizations (2.25%; *n* = 2). All studies were carried out in public schools/educational institutions participating in SFPs subsidized by the government and offered to the students for free and/or reduced-price meals.

Most studies (23.60%; *n* = 21) evaluated only one school. There was a range from 1 to 480 evaluated schools, except for six studies that did not report the number of schools. The most explored teaching stage was elementary school, in 63 studies (70.79%), followed by middle school, 43 (48.31%), high school (23.60%; *n* = 21), preschool (7.86%; *n* = 7), youth and adult education (YAE) (3.37%; *n* = 3), and daycare (1.12%; *n* = 1), isolated or together with the other stages. However, one study did not report the teaching stage.

Considering the sample of subjects who assessed acceptance of menus, all studies included students ranging from 10 to 35,393. More than half of the studies (74.16%; *n* = 66) evaluated responses from 10 to 1000 students, 19.10% (*n* = 17) between 1000 and 6000, and 3.37% (*n* = 3) included 14,717, 34,434, and 35,379 students, respectively. Nevertheless, three (3.37%) did not report the number of participants. In two studies, parents (*n* = 2044; *n* = 71) participated, and in three studies, teachers (*n* = 175; *n* = 1978) participated. The presence of cooks (*n* = 4) was verified in two studies, and food handlers and teachers (*n* = 5) in one study.

### 3.2. Methods of Evaluating the Acceptance of School Menus

The results showed that the most used acceptance assessment method was the hedonic/Likert scale of 2, 3, 4, 5, 6, 7, and 9 points, present in 62 studies (69.66%). The 5-point scale was the most prevalent (50.56%; *n* = 45). Mathematical formulas and/or visual estimates evaluating food consumption and leftover food (plate waste or rest ingestion) were used in 36 studies (40.45%). The qualitative methodology of collective subject discourse (CSD) evaluating acceptance was present in 1 study (1.12%), and questionnaires and/or interviews with objective and/or subjective questions were observed in 40 (44.94%) (Table 2).

For each study, methods were found singly or in combination. The most frequent combination was the hedonic/Likert scale associated with questionnaires and/or interviews in 21 studies (23.60%), then 20 (22.47%) studies with scale and mathematical formulas and/or visual estimation, 15 (16.85%) studies with mathematical formulas and questionnaires and/or interviews, and a combination of the three methods in 7 studies (7.86%). Only one study (1.12%) combined the qualitative methodology of collective subject discourse (CSD) and questionnaires and/or interviews. Finally, it is noteworthy that 44 studies (49.44%) used only one assessment method, as mentioned above.

The same method was used to evaluate different attributes, depending on the study. Regarding scales, the nomenclature “hedonic scale” was used by 37 studies (41.57%), “Likert scale” by 23 (25.84%), 5-point nongender horizontally oriented facial scale by 2 (2.25%), and both “hedonic scale” and “Likert scale” by 1 (1.12%). For the evaluation of leftover food, various mathematical formulas were used by direct weighing (33.71%; *n* = 30) or the visual estimation method (13.48%; *n* = 12). Of these, five Brazilian studies (5.62%) evaluated the meal repetition percentage/index by direct weighing. Questionnaires were applied in 38 studies (42.70%) and interviews in 4 (4.49%).

Table 3 presents the main sensorial and acceptance evaluation methods each country uses. Of the 13 countries, the hedonic/Likert scale was the most prevalent method or the only one used (61.54%; *n* = 8). Questionnaires and/or interviews were the most used by four countries (30.77%), and mathematical formulas and/or visual estimation by six (46.15%). Only one country used the qualitative methodology of collective subject discourse (CSD).

All countries used more than one method in their studies, except the Philippines and Spain. Among the countries that presented more than one study, the hedonic/Likert scale was used in all studies from South Korea (*n* = 13) and India (*n* = 2), in half of Brazil (*n* = 21) and Colombia (*n* = 1), in 6 (75%) from Italy, 9 (75%) from the United States and, in 1 (33.33%) from Chile. However, it was not present in studies from Spain. For countries with only one study, Ghana, Philippines, Paraguay, and Finland included the hedonic/Likert scale, and Georgia did not use it.

In general and for each country, the types of methods in the studies remained the same over the years. However, the use of one or more methods varied depending on the country or the author, as well as the nomenclature of the evaluated attributes and the forms of presentation of the methodological procedures of each study.

### 3.3. Main Menus/Meals Evaluated

For each study, meals were evaluated either alone or together. The most evaluated meal among the studies was lunch (61.80%; *n* = 55), followed by snacks (43.82%; *n* = 39), breakfast (6.74%; *n* = 6), and dinner (1.12%; *n* = 1). Among the studies that evaluated more than one type of meal, there were six studies for lunch and breakfast (6.74%), four for lunch and a snack (4.49%), one for lunch and dinner (1. 12%), and one for breakfast, lunch and dinner (1.12%).

In Brazil, it was found that all the served snacks consisted predominantly of sweet preparations (such as “snacks”) or salty preparations (such as “lunch”), served alternately during the week.

### 3.4. Risk of Bias

Among the analyzed studies, all (*n* = 89) had a low risk of bias. All studies evaluated the acceptance of school menus offered by SFPs around the world and answered the main research question (Table S3 in Supplementary Materials).

## 4. Discussion

Several studies (*n* = 89) used different methods for sensory evaluation and acceptance of school menus from school feeding programs (SFPs). Thus, the concern of the scientific community and the importance of the theme in the context of school meals is evident.

Considering the countries in which studies were included in this review, Brazil, South Korea, the United States, Italy, Colombia, India, Chile, Ghana, Spain, Paraguay, and Finland have established SFPs, as well as the Philippines [108,109]. For Georgia, no information was found about a national program. The fact that it does not have a program may have influenced the low number (*n* = 1) of studies in that country. Despite a national school feeding program in the mentioned countries, only Brazil is evaluating the acceptability of school meals in public schools mandatory [110]. For the other countries, this was not observed.

The most used acceptance method among the studies was the hedonic or Likert scale of 2, 3, 4, 5, 6, 7, and 9 points (69.66%; *n* = 62), and the 5-point scale was the most prevalent (50.56%; *n* = 45). The hedonic scales are classified as nominal, verbal, numerical, graphic, or mixed and express the taster’s likes or dislikes for a food product [111]. This method is the most used in the sensory analysis of food, as it is quick to perform, easy to understand and apply, and capable of measuring individual variations more accurately. Furthermore, it presents more attractive techniques, can be used with untrained tasters, and can evaluate many sensory stimuli [112].

The food industry widely uses hedonic scales, applied with consumers to obtain information that helps in decision-making for developing new food products to be introduced in the market, new variations, or reformulations [113,114,115,116]. Thus, hedonic or affective tests are often used to assess acceptance or optimize the acceptability of these products [117,118,119].

The verbal taste scale for testing children, known as Peryam and Kroll (P&K), is a 9-point hedonic scale that uses verbal anchors using the terms “super good” to “super bad”. After testing, the author found that the 9-point scale discriminated better than the 7-point among children aged 5 to 7. They have corroborated these results when comparing 3-, 5-, and 9-point scales with children aged 8 to 10 years. They disputed the hypotheses that facial scales were superior to verbal and that shorter ones were better than longer ones [120].

The authors of [120] pointed out that facial scales can confuse or introduce an unintentional bias, as a face representing “disgust” can be interpreted as conveying anger, and one intended to show “likes” can suggest “happiness”, rather than representing the child’s opinion about the food.

These authors have shown that responses are more likely to be crowded at the upper end of the scale when there are fewer response options (5-point hedonic scale). When using a 7-point hedonic scale among children aged 8 to 14, it was concluded that a vertical orientation leads to more positive responses than a horizontal orientation. As for the horizontal scale, the positive side on the left leads to higher ratings than a scale with the negative side on the left.

Due to the advancement of digital communication and the rapid popularization of emojis, interest in their application to understanding how consumers perceive and describe their experiences with food products has been aroused. In this sense, emojis can provide information about human behavior that cannot be obtained by analyzing communications only in written form [121].

Other authors evaluated the taste and emotional response of children aged 8 to 11 (3rd, 4th, and 5th grades) by applying a pictorial facial scale based on emojis. The results demonstrated a high positive correlation between emotional response and taste. For the authors, the emoji scale proved to be applicable for measuring emotional responses using names of verbal food stimuli with children in the United States. Therefore, the authors supported the choice of emojis for acceptance assessment, precisely the emotional response. None of the studies included in this review used emojis present in social media [122].

Of the 13 countries, the hedonic or Likert scales were the most prevalent method (61.54%/*n* = 8). The 5-point scale was the most used. The 7-point scale was used in most studies from Italy (37.50%), the Philippines (100%), and Finland (100%). In Brazil, despite not being the most prevalent method, 23 studies (54.76%) used a scale, of which 42.86% used 5 points (*n* = 18), 4.49% 3 points (*n* = 4), and 2.38% (1 study) used 3 and 5 points. As Kroll (1990) pointed out, 9-point scales are more adequate for studies with children than 3-, 5-, or 7-point scales; however, they were not prevalent in this systematic review.

Preferences are an essential indicator of food consumption, predicting the average amount consumed and the proportion of people who will accept these foods [123]. Therefore, studies evaluated different perceptions using the same method.

Acceptance is an experience characterized by a definite positive attitude of the subject about the analyzed object, which the actual use of a particular food can measure [45]. The acceptance of students is an essential factor in establishing the quality of the service provided by school food services regarding the provision of school meals [124]. It improves nutrient ingestion by students and reduces food waste, contributing to sustainability.

The Brazilian school feeding program constructed a manual to evaluate the acceptability of school meals. Acceptability is described as the set of methodological procedures, scientifically recognized, intended to measure the acceptance index of the food offered to students. It is part of the sensory analysis of food, which evokes, measures, analyzes, and interprets reactions to the characteristics of foods and materials as perceived by the organs of sight, smell, taste, touch, and hearing [124].

This Brazilian manual suggests using two methods: hedonic scale and rest ingestion. For the hedonic scale, the use of verbal or facial scales depends on the age of the students, but all of them use a 5-point scale. For the analysis of the answers, if the sample presented a percentage ≥85% in the expressions “I liked” (4 points) and “I loved it” (5 points), the tested food was accepted [124]. Even though this governmental manual suggests the use of hedonic scales, the majority of the studies from Brazil did not use it, nor did the other ingestion evaluations. This is because the manual was published in 2010, and there have been many studies before this year. Studies from Brazil published from 2013 onwards began to use the methods described in the aforementioned manual, using only the hedonic scale, the rest intake, or both. After the manual’s publication, only eight studies adopted different methodologies [12,74,78,79,82,86,89,92].

In the United States, the School Nutrition and Meal Cost Study of 2019 stand out, which evaluates student participation, satisfaction, and plate waste in volume 4. It is considered a nationally representative study with a sample of over 1000 elementary, middle, and high school students. Additionally, it investigated parental satisfaction [99], an important data collection strategy that can contribute to investigating the public’s opinion for decision-making and implementing public policies in this context. This national study used scales of 2, 3, 4, and 5 points because each scale evaluated different attributes. Another extensive school program that does not use 7- or 9-point scales is pointed as the best strategy.

The mathematical formulas and/or visual estimates evaluating food consumption and leftover food (plate waste or rest ingestion) were used in 36 studies (40.45%). Twelve studies (13.48%) employed the visual estimation method. This method determines the waste of dishes, indirectly measuring food waste. Therefore, it requires trained observers to estimate the weight of the waste. Depending on the study, it may be an efficient collection method [125].

However, even trained observers’ skills may vary in estimating the amount of food ingested and discarded, allowing this to be a source of bias. Additionally, observers who have not tried the food offered to the students may not understand the reasons for discarding it due to characteristics such as inadequate temperature and undercooked food [76].

Regarding the Brazilian studies, the acceptance of the menus was evaluated using the method “Visual Estimation of Rest on Each Plate”. The method aims to verify the amount of food offered to the student and not consumed, that is, left on each plate [56]. It is the most recommended due to its speed, ease of application, validity, and reproducibility of results, better reflecting individual variations [44,55].

The method of “Visual Estimation of Leftovers in Each Plate” is valid but limited since students who do not participate in the school feeding program are not included in the indices that determine the acceptability of school feeding due to the rejection of the menu and social constraints, among other reasons [55].

The visual estimation method through digital photography of meal trays before and after ingestion allows evaluators to have the time to consider the amount of food consumed by each student carefully. The author verified a 92% agreement between the weighed trays and those visually estimated from post-consumption photographs [80].

The evaluation of consumption and leftover food (plate waste or rest ingestion) using mathematical formulas was performed by twenty-four studies (26.97%). In studies in Brazil, the methods named “Measures of aggregate leftovers”, “Menu repetition percentage/index”, “Effective Attendance Index”, “Rest Ingestion index”, “Rejection Index”, “Adherence Index”, and “Acceptability Index” were used. In South Korea, “Examinations leftovers” was used. In the United States, “Plate waste” and “Aggregate plate waste” were used. In Italy, the “Satisfaction Index” was used. In Chile, “Real intake of food” and “Satisfaction Indicator” were used. In Spain, “Estimate of leftovers” was used, and in Paraguay, “Percentage of food consumption” was used.

Among them, the Brazilian studies [19,44,45,55,59] evaluated the acceptability of school meals by the percentage/index of repetition of the meal (direct weighing), that is, whether or not students repeated the offered meal. However, this calculation does not constitute a form of evaluation of acceptability since the student can practice repeating the meal to satisfy his physiological hunger and not necessarily because he appreciates/likes the food offered.

This method may present biases due to several factors. The training of the evaluators, the equipment and materials used (scales, spreadsheets, etc.), the data collection procedures used, the way of portioning the food offered, the type of mathematical formula applied and its cutoff points, and the interpretation of results, among others. Thus, comparing these formulas in terms of effectiveness and validity becomes difficult.

Questionnaires and/or interviews with objective and/or subjective questions were observed in forty studies (44.94%). Most respondents were children, mainly from the elementary school stage (70.79%; *n* = 63), generally comprised of the age group of 7 to 11 years.

Completely labeled response options help produce more reliable responses, as partially labeled ones require clear definitions of the offered response options [126]. Thus, dealing with partially labeled answer options becomes more challenging since their logical and abstract thinking is limited, and they must interpret and translate the unlabeled options themselves.

It is worth considering Piaget’s theory of child development to combine developmental skills and the cognitive demands of survey research [126]. During childhood and adolescence, the continuous development of functions related to language, literacy, and memory is observed, which potentially affects their ability to answer a research question well [127].

The simplicity and clarity of the questions are essential for the development of questionnaires, making them immediately recognizable to children. These have difficulties with ambiguous and vague words as they tend to interpret words literally. Thus, the unequivocal wording of the questions is essential to improve the quality of the research data [128].

Extensive questionnaires that seek detailed information should be avoided as they can lead to withdrawal and incomplete responses [129]. The content and context of the questions and the physical environment can affect children and adolescents and, consequently, the quality of the data [127].

The advantages of applying questionnaires include the speed of application, the ability to collect a significant amount of data, and the ability to reach large samples. However, the disadvantages are usually the low return rates and literacy levels [130]. Furthermore, the absence of a valid survey with tested questions makes confidence in the validity of the results questionable [80].

Only one study [12] used the qualitative methodology of collective subject discourse (CSD) proposed by [131]. The DSC is anchored in the theory of social representations, which, through open questions, collects and analyzes individual testimonies in empirical opinion polls, identifying key expressions (ECH) and central ideas (CI). Thus, extracts from different individual testimonies are used to elaborate collective testimonies. These are written in the first person singular, representing the collective opinion.

The DSC makes it possible to reach numerically more representative samples expressing the thoughts of a given population. The accurate collection of subjective data and contemplating quantitative aspects of studies across diverse academic areas of knowledge are required [132]. The researcher must pay attention to avoid the extinction of the less recurrent answers because an individual’s speech is unique [133].

Most evaluated meals in Brazil were snacks, consisting predominantly of sweet preparations (such as “snacks”) or salty preparations (such as “lunch”), served alternately during the week. This composition of snacks is a common practice in Brazilian states and aims to meet the nutritional needs of students. Among the savory preparations, rice and beans stand out, a food combination that is part of the country’s food culture. For Maciel (2004), their consumption goes beyond regional differences, social class, or ethnic origin, constituting the basic food of Brazilians. Respect for food culture is important for guaranteeing the human right to adequate and healthy food [134]. In other countries, lunch was the most rated meal and generally included main courses, side dishes, and desserts. So, some typical preparations were verified, depending on the country.

It is understood that the school menu preparations do not necessarily indicate that the student likes and is satisfied with the food offered. It can characterize a solution to their physiological need. Many students consumed school meals because it was the only alternative to eat during the class break due to the absence of canteens and/or cafeterias in the school environment [12].

Due to hunger and lack of food offered by the family, children consume the food provided at school even if it is not to their total liking or preference. In this way, it causes an increase in the acceptability indexes of the program’s meals, which do not reflect the actual acceptance of the students [55]. Other studies have reported that school meals represent the only daily meal for many students. In this way, it is a vital tool for fighting hunger [6,8,26,92,135,136,137,138].

Some studies found that younger students rated school meals more positively and satisfied than older students. This finding may be related to the fact that with increasing age, children and adolescents become more aware of their food preferences [139]. Thus, they are the most frequent consumers of school meals [45,59,62,78,81,98,140,141].

School meals are planned to ensure effective consumption and still be appreciated by students, one should consider and understand the existence of several factors involved in this process, which directly influence the food choices of these individuals. It is known that food choices developed during childhood are the result of subjective perceptions and opinions about food, verified through the five bodily senses: taste, smell, touch, sight, and hearing. Therefore, children consider the palatability and organoleptic characteristics of foods (taste, smell, texture, and appearance) as determinant aspects of their food preferences that influence the composition of their consumption patterns [21,50,142,143].

Intervening factors can be the long lines to receive meals, time restrictions to eat, lack of variety of preparations, and consumption of food purchased outside of school [27]. Overall, food choice is mainly affected by parents, other children, and children’s advertising [144].

Nonetheless, it is considered that, in addition to these listed characteristics, food choice is affected by interdependent factors, namely, physiological and nutritional needs, genetic predisposition, personality parameters, and sociodemographic and cultural aspects [144,145]. The environment in which the food is offered interferes with its approval in the construction of the eating habits of this population [19].

The children are more likely to prefer foods that are familiar to them over those unfamiliar. This familiarity comes from their experiences with food, and it is necessary to expose them several times to encourage consumption patterns that encourage the practice of more varied and healthier eating habits [146,147]. In this way, it becomes a challenge to achieve a balance between healthy eating and eating accepted by students [54].

Hence, efforts should be directed toward greater acceptance and adherence of students, based on the verification and evaluation of their indices, as these act as necessary devices that, when measured through specific methods, allow assessment of the quality of school meals provided by schools [148]. Moreover, they avoid wasting food and public resources when purchasing rejected foodstuffs [149].

This review has some limitations. Some studies, written in a language other than English, were translated through a translation platform. Therefore, some information may have been lost due to language barriers. Additionally, there is a wide variety of methods to assess the acceptance of school menus, many of which have their evaluation parameters. Thus, comparing them in terms of validity and efficacy is a relevant limitation.

## 5. Conclusions

This systematic review found many studies that used methods to assess the acceptance of school menus offered by SFPs in different countries. Brazil, South Korea, and the United States were the countries that most investigated this issue, probably because they have the largest SFPs, considering the time of existence and coverage. Only in Brazil is evaluating acceptability mandatory. The most prevalent evaluated meal was lunch since most students go to school during the day and spend morning and afternoon studying. Moreover, the most evaluated teaching stage was elementary.

About 70% of the studies used the hedonic or Likert scale, with a higher prevalence for the 5-point scale. Thus, it is the most widely used method, probably due to its low cost, simplicity, ease of elaboration, speed of application, and greater possibility of student understanding. Furthermore, it presents more attractive techniques, can be used with untrained tasters, and can evaluate many sensory stimuli. It is a method already used by the industry to develop or reformulate products.

However, it is essential to know the study’s target audience, establish the best application method, and if it will evaluate the complete meal or isolated dishes. When using the hedonic scale to evaluate the acceptability of a whole meal, schools may have difficulties understanding which preparation in a meal brought the results closer to acceptability or rejection. When evaluating leftovers, it can be easier to identify the most rejected dishes.

Some authors state that facial scales can confuse or introduce an unintentional bias, as a face representing “disgust” can be interpreted as conveying anger, and one intended to show “likes” can suggest “happiness”, rather than representing the child’s opinion about the food. They support the choice of emojis for acceptance assessment; however, none of the studies included in this review used emojis.

The method of mathematical formulas and/or visual estimation evaluating consumption and leftovers, despite being used by about 40% of the studies, is difficult to compare due to the different mathematical formulas used for the evaluation. In addition, some studies did not fully describe the data collection procedures, which may compromise the reliability of the results. Therefore, despite being a good way to evaluate preferences for a meal, studies must fully describe all the necessary steps for reproducibility in further studies.

Questionnaires and/or interviews with objective and/or subjective questions and the qualitative methodology (collective subject discourse) must be adapted to the stage of cognitive development of each age group to achieve their goals of being applicable and understandable. Therefore, they were less used in the studies because researchers look for methods that can be applied to different age groups.

Further studies involving numerical scale methods adapted to the target audience are necessary. Thus, it will be possible to continuously evaluate the menus or foods provided by SFPs and compare scientific data worldwide. In this way, macro and micro policies can be developed so that the school community served by these programs has an adequate consumption of the food and meals offered, contributing to the integral development of students.

## Figures and Tables

**Table 1:** Main descriptive characteristics and results from the included studies.

Reference, Year, and Country	Objectives	School Sample (SS) Teaching Stage (TS) Participants (P)	Sensory Evaluation and Acceptance Methods Performed	Evaluated Attributes	Results
Head et al. (1977) USA [39]	To measure the acceptability of school-served food items by three methods and evaluate the relative usefulness of each	SS: *n* = 13 TS: Elementary, middle, and high school P: Students (*n* = 240)	Three methods Form with: 5-point hedonic scale “How you liked it?” (“great” = 5, “good”, “OK”, “not very good”, or “terrible” = 1) 5-point scale “How much you ate?” (“all” = 5, “most”, “about half”; “just tried it”, or “none” = 1) Mean weight = weight of 4 served trays PW = from every 2nd, 3rd, or 4th student who had completed scales. Separated by individual food items from every 25 students (pooled and weighed) Estimate of the amount consumed = mean plate waste − mean amount served (for each item)	Acceptability Hedonic rating scale (HED) Amount consumed rating scale (AMT) Plate waste	The reliability of the HED scale was highly significant, and the AMT scale was significant for all but one item. The interaction of HED and AMT scores was insignificant, implying that liking or not liking had little effect on how students reported how much they had eaten. The analysis showed a more positive relationship between the AMT score and food consumption than the HED score and consumption. A statistical model was constructed for predicting food consumption from ratings; it was possible to obtain an indication of consumption by elementary students from either scale.
Devan et al. (1988) USA [40]	To assess the influence of vegetable preparation training on the amount of vegetable plate waste; to compare student ratings of cooked vegetables before and after training; and to determine if quality characteristics, as measured by a professional sensory panel, are affected by training in proper vegetable preparation techniques	SS: *n* = 5 TS: Elementary school P: Students (from 37 to 147)	5-point hedonic scale (“great”, “good”, “so-so”, “bad”, and “awful”) 3-point hedonic scale (“hot”, “just right”, and “too cool”); (“too much”, “right amount”, and “too little” Visual estimate Portion of broccoli remaining: “full portion” (4) to “none remained” (0)	Sensory quality Flavor and appearance of broccoli Temperature and amount of broccoli Plate waste	The average scores referring to the sensory quality of broccoli for schools A, B, C, D, and E were flavor (2.9; 2.43; 2.57; 2.89, and 2.84), appearance (2,14; 2.26; 2.29; 2.40, and 2.51), temperature (1.49; 1.57; 1.57, 1.68, and 1.83), and portion size (2.42; 2.48; 2.22; 2.05, and 2.24), respectively.
Stalls (1997) USA [41]	To determine the level of acceptability of three different low-fat brownies compared to the USDA brownie recipe	SS: All schools in the school district (*n* = not informed) TS: Elementary school P: Students (*n* = 77)	9-point hedonic and pictorial face scale “super good” (9), “really good” (8), "good" (7), “just a little good” (6), “maybe good or maybe bad” (5) to “super bad” (1)	Sensory evaluation Consumer preference or acceptance/likability testing (to measure how much 4th-grade students like, prefer, or accept)	The average flavor score for the regular brownie was 7.2. Most students (83.11%, *n* = 64) rated the brownie between “super good” (9) and “just a little good” (6), while 11.68% (*n* = 9) judged it as “just a little bad” (4) to “super bad” (1).
Kim and Kim (1997) South Korea [42]	To contribute to improving the quality of school lunches by analyzing the satisfaction of school lunches provided in schools for children of meals in Seoul, a large city, and Gangneung, a small city	SS: *n* = 28 TS: Elementary school P: Students (*n* = 3.590)	5-point Likert scale (1 = ”very dissatisfied” to 5 = “very satisfied”) % of students who had left leftover food (by grade and food type) (“food is not warm”, “disliked food”, “the food is tasteless”, “have no appetite”, “cooking was not done properly”, or “too much”)	Satisfaction (appearance, taste, temperature, texture, and overall satisfaction) Examination of leftover food (type of food left: rice, soup, kimchi, side dish, and milk) Reasons to leave food	The general satisfaction regarding the type of food was: rice (3.90 and 3.78), soup (3.70 and 3.64), side dishes (3.79 and 3.66), and milk (2.60 and 2.53) for boys and girls, respectively. The mean leftover was higher for soup and lower for rice. The major reasons for leaving leftover were “dislike the food” (27.4%), “too big portion size” (23.6%), “food is not tasty” (19.1%), “low appetite” (17.6%), “food is too overcooked or undercooked” (3.9%), and “food is not hot enough” (3.2%).
Baxter et al. (2000) Georgia [43]	To estimate the relationship between fourth-grade children’s consumption and preferences for school lunch foods through observation	SS: *n* = 4 TS: Elementary school P: Students (*n* = 237)	Eaten amounts observed (coded as none = 0.0, taste = 0.1, some = 0.25, half = 0.5, most = 0.75, all = 1, and >1 serving = 2) Interview (audio-recorded and transcribed) Students were asked what they ate for school lunch (free and non-suggestive prompted recall) and whether they liked foods observed and/or reported eaten “not at aIl”, “a little”, or “a lot” (coded as 0, 1, and 2, respectively)	Children’s observed consumption Preferences for school lunch foods	Results indicated a significant relationship between observed consumption and preferences (*p* < 0.001); as preferences increased, consumption also increased. Consumption (least squares means with standard error in parentheses) was 0.11 (0.04) servings for foods liked “not at all”, 0.54 (0.03) liked “a little”, and 0.92 (0.02) liked “a lot”. All other main effects and interactions with preferences failed to reach statistical significance (all *p*’s > 0.14). Thus, the children eat virtually all of what they like “a lot”, about half of what they like “a little”, and almost none of what they like “not at all” during school lunch.
Brandão (2000) Brazil [44]	To evaluate the acceptance, preference, percentage of adherence, opinions, and expectations of students from 1st to 4th grades, regarding the menus of the school lunch program of municipal schools in the city of Campinas between 1997 and 1999	SS: *n* = 10 (1st step) *n* = 4 (2nd step) TS: Elementary school P: Students (*n* = 5.407: 1st step) (*n* = 384: 2nd step)	Average acceptance % = 100 − [(T_0_ × 0) = (T_25_ × 25) = (T_50_ × 50) = (T_75_ × 75) = (T_100_ × 100)]/T_0_ + T_25_ + T_50_ + T_75_ + T_100_ Weight of prepared food = W × H × Hp/100 (P = specific weight of the prepared food, A = area (diameter) of the cooking pot, Hp = height reached by the food in the pot) % of average acceptance = prepared − clean leftovers − leftovers × 100/prepared − clean leftovers, that is, the total weight of the lunch consumed × 100/total weight of the distributed lunch % of adherence = *nº* of students who joined the SMP/*nº* of students present (on the day and period) × 100% % of average repetition = number of students who repeat the SMP menu/number of students who adhered × 100% Adherence (Questionnaire I) Questions (*n* = 6) or Non-adhesion (Questionnaire II) Questions (*n* = 4) Open-ended, closed-ended questions Facial structured hedonic scale (5-points)	Acceptance and preference (1st step) Visual estimate (VE) of leftovers on each plate Amount of food left on the plate (0%, 25%, 50%, 75%, or 100%) Measures of aggregate leftovers (MAL) Program adherence percentage Menu repetition percentage Adherence and non-adherence (2nd step) The type of snack consumed and the opinion Acceptance	The average acceptance of all menus, obtained by the VE and MAL methodologies, indicated that the levels were highly satisfactory, ranging from 88 to 94% of acceptance. As for the % of adherence, some schools had very low levels, and it varied between 22.41 and 71.09%. The average percentage of repetition was 25%, and only two schools did not allow repetition. In the second stage, students who joined and did not join the program listed which types of food they would choose to include in school lunches and presented very similar food choices. Some foods were already on the menus, such as fruits, rice with meat, and soups, and some mentioned nutritious foods, such as leafy and non-leafy vegetables, rice and beans, and sandwiches, notably hot dogs. The menu’s average values of acceptance, both those who adhered and those who did not, were between 3.0 and 4.0, that is, between “neither liked/nor disliked” and “liked” on the scale.
Sturion (2002) Brazil [45]	To evaluate the performance of the school feeding program in municipalities in different regions of Brazil, with different management characteristics	SS: *n* = 20 TS: Elementary, middle P: Students (*n* = 2.663)	% of acceptance = weight or volume of the preparation consumed/total weight or volume of the preparation × 100 % of repetition = *nº* of students who repeat the menu/*nº* of students who joined the program × 100 IAE = total number of students effectively served/total number of students enrolled × 100 Questions (*n* = 5) “Do you usually eat the lunch offered at school?” (“yes” or “no”); “How many days a week?” (1 day/wk, 2 or 3 days/wk, 4 or more days/wk); “Name up to 5 foods that you most and least like to eat in school lunches” (*n* = 2); “If you don’t eat the lunch offered at school, write the reason”	Acceptance (measures of aggregate leftovers) Repetition percentage Adherence effective attendance index (IAE) High (>70%), medium (50 to 70%), low (30 to 50%), and very low (<30%). Custom and weekly frequency of consumption	The average rate of acceptance of the menus, obtained from the method of “Measures of Aggregated Leftovers”, is around 85%, broken down by school unit, the highest index being 97.0% and the lowest 72.9%, with significant differences between schools. The repetition rate is linked to the availability of leftover food. Thus, it cannot be adopted as an indicator of acceptance of the meal. The average rate of total adherence based on the effective attendance index (IAE) was low (45%), the highest being 88.3% and the lowest being 18.6%. As for the habit and frequency of snack consumption, only 46% did it daily. The highest is 81.0% by a school, and the lowest is 15.2%.
Pagliarini et al. (2003) Italy [46]	To set up an evaluation card and a procedure to test the grading of meals supplied to school refectories by the Municipality of Milan	SS: *n* = 2 TS: Elementary school P: Students (*n* = 88)	7-point hedonic facial scale Super good (7), really good (6), good (5), maybe good or maybe bad (4), bad (3), really bad (2), and bad (1)	Acceptability “Appreciated” ≥4	As for the averages of acceptability observed from schools’ R and L: first course (4.41; 5.02), second course (4.62; 5.15), vegetables (4.19; 4, 87), and fruits (4.93; 5.46), respectively. For R and L schools, the most appreciated first dishes were: pasta with bolognese sauce for both (5.39; 6.00). Second course: roast chicken for both (6.23; 6.50). Vegetables: baked potato for both (5.97; 6.08). Fruits: orange (5.58 for R). The least liked first course: buttered pasta (3.34) and vegetable puree (3.87). Second course: potato omelet for both (2.92; 3.72). Vegetables: carrots (2.83) and red salad (2.78). Fruits: pear (4.19), respectively. For school L, no significant differences were found in fruit preferences. There were no significant differences in preparation between the two grades and schools.
Hong and Chang (2003) South Korea [47]	To identify the attributes of food and service quality, to examine the levels of satisfaction and plate wastes leftover in school meals, and to determine the relationship between student satisfaction and plate wastes	SS: *n* = 11 TS: Elementary school P: Students (*n* = 999)	5-point Likert scale (1 = ”not at all”, 2 = ”no”, 3 = ”normal”, 4 = ”yes”, or 5 = “very much”) 6-point Likert scale (1 = ”never eating”, 2 = ”eating little”, 3 = ”eating a quarter”, 4 = ”eating a half”, 5 = “eating three quarters”, or 6 = ”eating all”) (“too much”, “having no appetite”, “disliking foods”, “bad taste”, “undercooking/overcooking”, or “too often provided foods”) 5-point Likert scale (1 = ”not at all”, 2 = ”no”, 3 = ”normal”, 4 = ”yes”, or 5 = “very much”)	Satisfaction Food (preference, variety of menu, taste, appearance, temperature, usage of seasonal food, nutrition balance, food safety, serving size) Food intakes (for cooked rice, soups, kimchi, meats, fish, vegetables, and dessert) Reasons for leaving foods Satisfaction scores by eating habits (“leaving foods” or “eating all foods”)	The satisfaction score with the foodservice quality for the food-related factors was “average” (mean 3.20). The highest satisfaction was for the “nutritional balance” attribute (3.85) and the lowest was for “appearance” (2.87). The intake rates of soup, kimchi, fish, and vegetables were lower than those of other foods. Regarding the relationship between satisfaction and food leftovers, it was observed that the group “eating all foods” had significantly higher satisfaction scores with the meal than the group “leaving foods”. Rice was the type of food most left by the students, and the main reasons were “too much” and “having no appetite”.
Flávio et al. (2004) Brazil [48]	To determine the chemical composition and acceptance of the lunch offered to the elementary school students of public school in Lavras, MG, and to verify if they meet the objectives of the School Feeding National Program	SS: *n* = 1 TS: Elementary, middle P: Students (*n* = 598)	Questionnaire Questions: objective (*n* = 4) (yes or no; yes or no; (does not consume, 1x/wk to 5x/wk) and Subjective (*n* = 2) (more or less preferred menus and the menus that students repeated the most or not)	Preference and acceptance	Most of the students (72%) had the habit of consuming the lunch offered by the school. Regarding the weekly frequency of consumption, 25% consumed daily, and 16% did not. The main reason was not feeling hungry when the lunch was distributed. A total of 61% of students had the habit of repeating their school lunch. As for students’ preferences, rice seasoned with ground beef had the highest percentage of choice (90%), and corn flour soup with eggs and cabbage had the lowest (27%).
Martins et al. (2004) Brazil [49]	To evaluate the acceptance of school meals in the elementary public schools of Piracicaba/SP that benefited from the National School Meal Program	SS: *n* = 12 TS: Elementary, middle P: Students (*n* = 480)	Acceptability = average acceptance, rejection, and adhesion rates for the preparations served % of acceptance = total weight of food distributed/total weight produced to serve the clientele Total weight produced = average weight of the portion served × *nº* of portions served + leftovers (food not distributed) % of wasted meal = weight of food distributed/weight of food discarded (served and not consumed) Adherence rate = % of students who joined the meal/total number of enrolled students present on the assessment day Questionnaire Questions (*n* = 4) (“Do you usually eat the lunch offered at school? If so: “which dishes do you like the most?” If no: “why don’t you eat it?” “which dishes do you like least?”)	Acceptability Acceptance index aggregate leftovers method “High” = 90% Rejection index waste = values greater than 10% Adherence index “High” (>70%), “medium” (50 to 70%), “low” (30 to 50%), and “very low” (<30%) Reasons for adhering to school meals and more and less acceptable preparations	The results showed reasonable acceptability. Despite the high acceptance of some meals (above 90%), adherence to the preparations served at students’ entrance and recess time is “very low” (lower than 30%) and “low” (40 to 50% approximately), respectively. The rejection numbers found were close to expectations. The main reason for not eating school lunch was not liking the food (48%). Despite this, 67.7% of those who consume school lunches said they liked the food, it being the main reason for consuming them. The dishes they liked the most were pasta with meat (22%), chicken risotto (20%), and rice and beans (19%), while the ones they liked least were soups (47%).
Pagliarini et al. (2005) Italy [50]	To evaluate liking for meals supplied to primary school refectories of the Municipality of Milan	SS: *n* = 1 TS: Elementary school P: Students (*n* = 120)	7-point hedonic facial scale Super good (7), really good (6), good (5), maybe good or maybe bad (4), bad (3), really bad (2), and bad (1)	Acceptability “Appreciated” = >4 pts	From the age classification (7 to 10 years old), the average acceptability score was: 5.43, 4.90, 4.31, and 3.88 (first courses); 5.42, 5.18, 4.94, and 4.76 (second targets); and 5.15, 4.49, 4.13, and 4.03 (vegetables), respectively. The most preferred were risotto with pumpkin (samples B and P), roasted pork loin (C, T, U, and Y), and green salad and carrots (sample L). The most disliked were barley soup (D), cheese (A), and boiled zucchini (D and I) for the first courses, second courses, and vegetables, respectively. Unlike the others, the 7-year-olds provided increasingly higher acceptability scores than the intermediate score (4). Fruits/desserts obtained mean scores above 4 points and homogeneous preferences regardless of age, with significant differences only for apples and pears.
Lee and Lyu (2005) South Korea [11]	To evaluate the students’ satisfaction with the quality of middle school food service in the Busan area	SS: *n* = 8 TS: Middle school P: Students (*n* = 788)	5-point Likert scale (1 = “never important”, “very bad” to 5 = “very important”, “very good”)	Satisfaction (Gap = performance − importance) Meals (factor 1) taste, seasoning, temperature, a combination of main and side dishes, appearance, and portion size Sanitation (factor 2) Sanitation of meals Menu (factor 3) Dessert supply, event meal supply, variety of menu, and consideration of preferences in the menu	As for meals (factor 1), the average score for importance was 4.12, the highest for taste (4.57), and the lowest for appearance (3.40). For performance, an average of 3.05, with the highest for main course combination and side dishes (3.30) and the lowest for portion size (2.73). The sanitation of meals (factor 2) received 4.80 and 2.96 for importance and performance, respectively. The menu (factor 3) obtained an average of 4.15 for importance, the highest for variety of menu (4.28), and the lowest for consideration of preferences in the menu (4.00). As for performance, it presented an average of 2.91 pts, higher for variety of menu (3.19) and lower for consideration of preferences in the menu (2.84). Thus, the average scores (gap) were −1.11 (meals), −1.84 (sanitation), and −1.23 (menu), indicating that satisfaction with school meals was low.
Yoon et al. (2005) South Korea [51]	To determine the relationship between the students’ levels of involvement in school lunch service and their satisfaction levels with the service	SS: *n* = 14 TS: Elementary school P: Students (*n* = 1.254)	5-point scale (1 = “strongly disagree” to 5 = “strongly agree”) Affirmations (*n* = 5) “School lunch is what I need”, “School lunch is important to me”, “School lunch is valuable to me”, “School lunch gives me pleasure” (I like it), “I am interested in school lunch” 5-point scale (1-“strongly disagree” to 5 = “strongly agree”) FS = delicious food, is what I prefer, size of food is good for eating, a variety, well presented, temperature appropriate, fresh food each season, portion sizes appropriate SS = served food is clean and sanitary	Students’ level of involvement in school lunch service Satisfaction levels with the service Food satisfaction and sanitation satisfaction	The level of children’s involvement in the school lunch service was 3.06 points, indicating a moderate level. As for satisfaction, the “food satisfaction” factor obtained an average of 3.34. With the highest score for “food served is what I prefer” (3.61) and the lowest for “size of food is good to eat” (2.91). The food served is clean and hygienic, averaging 3.12, indicating moderate satisfaction. Thus, the school lunch service involvement positively correlated with food satisfaction.
Jang and Kim (2005) South Korea [52]	To provide basic information for satisfaction degree for school lunch program of elementary school students in Yongin city	SS: *n* = 1 TS: Elementary school P: Students (*n* = 646)	5-point Likert scale (“very satisfied” to “very unsatisfied”) 5-point Likert scale (“very insufficient” to “very sufficient”) 4-point Likert scale (“after 1st class”, “after lunch”, “bring home to drink”, or “do not drink”)	Degree of satisfaction Sufficiency of the amount of food provided Time of supplied milk intake	As for satisfaction with school meals, 24.7% of students were “very satisfied”, 36.8% “satisfied”, and 31.1% “fair”, representing 92.6% of the total number of students. There was no significant difference, but male students were more likely to be satisfied with their meals than female students. The amount of food offered was “very sufficient” (4%), “sufficient” (21.4%), and “fair” (64.9%), totaling 90.3% of the students; the most (70.6%) consumed milk after 1st class.
Lee and Jang (2005) South Korea [53]	To survey students in Gangwon province’s general opinion and satisfaction with the school food service programs implemented in Gangwon province	SS: *n* = 30 TS: Elementary, middle, and high school P: Students (*n* = 1.500)	Questionnaire Questions (*n* = 3) Answer options (*n* = 2) Questions (*n* = 2; 2) Answer options (*n* = 5; 7), respectively Questions (*n* = 1; 1) Answer options (*n* = 9; 4), respectively 5-point Likert scale (5 = “very satisfied” to 1 = “very dissatisfied”) T&N = overall taste; overall saltiness; diversity of rice, soup, and dishes; diversity of fruits, well-balanced nutrition, frequency of providing unfavorable foods, the satisfaction of the overall food categories SC = sanitary conditions of food	General opinion Food portion (quantity) Reasons why the food portion is not enough and the food is leftover Complaints of the food service and uncomfortable Satisfaction (taste and nutrition, sanitary conditions)	Regarding general opinion about SFP, portion sizes were “appropriate” for 70% of students. The main reason the portion was insufficient was “lack of side dishes”, and why the foods were leftover was “no tastes”. The main complaint was the “taste”, and the main uncomfortable issue was “many unfavorable menus”. As for student satisfaction with the taste and nutrition of school lunches, the “overall satisfaction with food” was 3.21, above average. Satisfaction with the “sanitary conditions of food” was 3.12.
Stroebele et al. (2006) USA [54]	To evaluate student acceptance of popular school lunch items that are reduced in fat and energy density	SS: *n* = 4 TS: Elementary school P: Students (*n* = average of 1.200)	5-point hedonic facial scale (ranging from an upset face for “bad” perceptions to a happy face to indicate a “good” rating)	Acceptance taste and appearance	All traditional preparations (pizza, french fries, and chicken fingers) had an average acceptance above 4.5 pts for the attributes of taste and appearance.
Pecorari (2006) Brazil [55]	To propose changes in the Municipal School Meal Program’s menu, from an elementary study in four of Piracicaba’s public schools and valuation of food supply and the conditions of production of the meals	SS: *n* = 4 TS: Elementary school P: Students (*n* = 2.256)	% of adhesion = *nº* of students who joined the PMAE/*nº* of present students × 100 % of average repetition = *nº* of students who repeat the PMAE menu/*nº* of students who adhered × 100 Visual estimation of leftovers on each plate Average acceptance % = 100 − [ (T0 × 0) + (T25 × 25) + (T50 × 50) + (T75 × 75) + (T100 × 100)]/T0 + T25 + T50 + T75 + T100 Facial structured hedonic scale (5 points) (“I like a lot” to “I dislike a lot”)	Adherence Repetition percentage Acceptance Categorical scale: 1 (0–25%), 2 (26 50%), 3 (51–75%), 4 (76–100%) Acceptability index = ≥85%	Mean food adherence for meals was low (39.32%). Per the school, adherence and percentage of repetition were 19.77 and 10.06%, 63.39 and 12.18%, 25.77 and 20.82%, and 48.37 and 26.38%, respectively. As for the acceptance of the visual leftovers estimate (consumption) methodology, the average of students who consumed 100% of the meals was 82.83%. By school, 81.67, 77.72, 88.43, and 83.51%. Acceptance of the structured facial hedonic scale methodology revealed the average of those who liked it a lot (29.16%) and a little (41.71%). Already 9.68% disliked it, and 1.05 disliked it a lot.
Flávio (2006) Brazil [56]	To evaluate the school meals (SM) offered to students in municipal (UEM), urban (EU), and rural (ER) school units in Larvas, MG, free of charge, due to the financial transfer of the National School Meal Program, regarding chemical composition, acceptability, adhesion, habits, and preferred preparations	SS: *n* = 16 TS: Elementary, middle P: Students (*n* = 835)	Acceptability test Amount of uneaten food left on each plate Average acceptance %: 100 − Σ of Total × %/total = X 100 − X = % of Acceptance Do you normally consume? (“yes” or “no”) How often do you consume? (“5x/week” for “none”) How do you rate the meals offered? (“excellent”, “very good”, “good”, “regular”, “bad”, “don’t know”) and justify; Among the snacks offered, do you prefer (“sweet”, “salty”, or “no preference”); Do you usually repeat your lunch? (“yes” or “no”). If yes, what is the menu?. Which of the meals served do you like the most? (free answer)	Acceptability Method of visual estimation of leftovers on each plate 0%: (ate all), 25%, 50%, 75%, 100% (just tasted) Acceptability ≥ 85% Adherence (consumption frequency) Non-adherence (did not consume), poor (1 to 3x/week), and strong (4 to 5x/week) Food preferences and habits regarding school feeding	Of the 112 analyzed preparations, the majority (*n* = 108) showed adequate rates of acceptance (>85%). Among them, 23 presented a percentage of acceptance of 100%, especially those with bread (as the main ingredient) added with different types of sauce or margarine and served with juices. As for average adherence (frequency of consumption), poor adherence (up to 3x/week) was observed by 76.5% of students, strong adherence (>4x/week) by 20.1%, and non-adherence by 3.4%. Thus, overall adherence to the program was poor. The reasons for consuming the food were: to like the offered preparation (62.5%) and to feel hungry during recess (23.7%), while for not consuming it the reasons were not liking it (1.6%), and to bring it from the house (1.0%). The majority (48.4%) rated the preparations as “good”, 31.6% (“very good” or “excellent”), and 16.6% (“fair” or “poor”). A total of 50.4% of the students reported having no preference in terms of taste, 27.2% preferred preparations with a salty taste, and 22.4% preferred sweet. Already 64% had the habit of repeating the feeding. Preparations containing rice as a primary ingredient, followed by pasta, wheat flour, corn, and milk, showed the highest percentages of choice, with 89.7%, 34.1%, 30.4%, 27.4%, and 19.2%, respectively, and seasoned rice was the most preferred (58%).
Rossi et al. (2006) Italy [13]	To assess the actual intake of food and nutrients in two elementary school classes and compare these intake values with the theoretical amounts established by the LARN (Recommended Energy and Nutrient Intake Levels)	SS: *n* = 1 TS: Elementary school P: Students (*n* = 36)	Satisfaction index = ratio between the amount of food consumed by the whole class and that distributed at the beginning in the individual dishes Value 1 (“consumed”) and value 0 (“rejected”) If rejected and consumed by the companion, values 0 and 2, respectively Satisfaction index = daily average per class	Food preferences (expressed by the satisfaction index)	Only the risotto and pasta with tomato (first course) and chicken nuggets (second course) had a satisfaction index equal to or greater than 1. That is, the average amount ingested was higher than that distributed. Vegetable soup (0.72) and fish (0.50), the first and second courses, respectively, had the lowest rates. All preparations (dishes) of side dishes and fruits obtained a satisfaction index lower than 1.
Byun and Jung (2006) South Korea [57]	To investigate the preference and satisfaction on the menu of school food service of high school students	SS: *n* = 10 TS: High school P: Students (*n* = 637)	5-point Likert scale 1 (minor) to 5 (major) 5-point Likert scale (1 = ”very dissatisfied” to 5 = “very satisfied”)	Preference Satisfaction	The average preference score for main dishes was 3.78, with the highest for sandwiches (4.14) and the lowest for boiled barley (3.33). Regarding side dishes, it obtained an average of 3.47, higher for steamed pork rib and seasoned roast chicken (4.30) and lower for spinach soybean soup (2.68). For dessert dishes, it was 3.98, higher for yogurt (4.16) and lower for currant tomato (3.86). The average satisfaction on the menu of school food service was 3.22, higher for beef rib soup (3.65) and lower for steamed mideodeok (seafood) (2.49). Overall, students showed high preference and acceptance for meat and sweet foods, while vegetables and tough and hard-to-chew menu items showed low preference and satisfaction.
Muniz and Carvalho (2007) Brazil [58]	To analyze the adherence and acceptance of school food and its determinants from the viewpoint of those who benefit from the program	SS: *n* = 10 TS: Elementary school P: Students (*n* = 240)	Questionnaire 15 closed questions (with spaces for justifications) and 5 open questions, including a request for the elaboration of a sentence about the school lunch	Adherence, acceptance, importance, quantity, variety, temperature, and most and least accepted foods	The program is considered important by 87% of students, and the main reason was need/hunger (41%). Regarding adherence, 33.5% always eat school meals, and 57.3% sometimes eat because it is not always what they are used to eating. The main reason for non-adherence is the inadequacy of preparation for their eating habits (41.5%). As for acceptance, 82% reported liking the food mainly because of the pleasant taste (76.7%), while 5.4% reported not liking it but eating it. A total of 79.8% consider the amount good/satisfied, varied (92.9%), and good temperature (85.8%). Among the foods they liked the most, cookies were the most cited (40.5%), and soups were the ones they liked the least (31.7%).
Conrado and Novello (2007) Brazil [10]	To evaluate the acceptance and nutritional value of school lunches offered to students	SS: *n* = 2 TS: Elementary school P: Students (*n* = 353)	Acceptance test (“liked the snack a lot”, “liked the snack”, or “did not like the snack”)	Acceptance	The average total acceptance for all preparations was 78.7% (including the options “liked” and “liked a lot”). The snacks that had the best acceptance were rice with meat and vegetables (95.2%) and beans, rice, meat, and salad (94.6%), whereas the ones with the lowest acceptance were pudding (57.5%) and rice pudding (59.7%).
Danelon (2007) Brazil [59]	To evaluate the management models (self-management and outsourcing) of the school food program (SFP) in Piracicaba (SP) and to identify the main changes imposed on the SFP due to the lengthening of the class period (full-time)	SS: *n* = 2 TS: Elementary, middle P: Students (*n* = 218)	Questionnaire Do you usually consume the lunch served at school in the morning break? (“yes”, 1 day/week, 2 days/wk or “no”); Say how many times a week (3 days/week, 4 days, or 5 days (every day); Main reasons to eat (free answer with 3 blank spaces); Do you repeat this meal? (“yes” or “no”); Main reasons not to eat (free answer with 3 blank spaces). The same questions for lunch and afternoon snack. What foods/meals do you like the most?; Which ones do you not like?; Which meals would you like to be on the menu more often?; What foods/meals would you like the school to offer on the menu? (free answers with 5 blanks) % of adherence = *nº* of students who joined the program/*nº* of students present × 100 % of repetition = *nº* of students who repeat the menu/*nº* of students who joined the program × 100 Average acceptance % = prepared quantity − clean leftover − tailings/prepared quantity × 100	Adherence Adherence Repetition percentage Acceptance Acceptability index ≥ 85%	Adherence (average) to school meals was morning snack (81.7%), lunch (95.9%), and afternoon snack (84.9%), with daily adherence frequency (5 days/week) in the morning snack: School A (45.6%) and B (61.2%), lunch: A (48.7%) and B (67.9%), and afternoon snack: A (43.3%) and B (62.9%). The main motivation for joining the morning snack was “hunger/want” (A = 44.6% and B = 30.8%), at lunch “hunger/want” (A = 50.6% and B = 36.9%) and in the afternoon snack “hunger” (A = 39.4% and B = 27.9%). The main motivation for refusal was “dislikes the food and preparations served” (A = 58.3% and B = 54.5%), “dislikes some preparations” (A = 41.7% and B = 50, 0%), and “dislikes food” (A = 45.5% and B = 41.7%), respectively. The preferences were for savory preparations (rice, beans, meat, and salad). The dishes not appreciated were scrambled eggs (A = 29.3%) and salads (B = 16.8%). The foods the students would like to be included on the menu were juices (A = 39.5%) and pasta (B = 25.5%). The percentage of repetition was 21.16%. The average acceptability for all meals was 89.83%, above the recommended (85%).
Park and Jang (2008) South Korea [60]	To investigate the satisfaction of fifth-grade (*n* = 264) and sixth-grade (*n* = 117) students from school food service in five elementary schools in Won-ju	SS: *n* = 5 TS: Elementary school P: Students (*n* = 381)	Questionnaire Questions (*n* = 12) 5-point Likert scale (1: “very poor” to 5: “very good”) Dissatisfied school food service factors = % of students dissatisfied for each reason, with response options (*n* = 5) or “others” % of students for each reason, with response options (*n* = 5) or “others” Type of food most likely to be leftover, with response options (*n* = 3) and reasons (*n* = 6) or “others” % of students, with response options (*n* = 6) or “others”	Satisfaction Overall satisfaction, quality (taste, flavor, temperature, and nutrition), quantity (steamed rice, side dishes, and dessert), meal composition (various kinds of food, *nº* of side dishes, use of seasonal food), food hygiene, and reflection of students’ opinions Dissatisfaction with the overall satisfaction with school meals (taste, quantity, hygiene, and temperature) Requirement for correction of school meals Food leftovers and reasons Change of attitude of students using the school food service	The average total satisfaction with school feeding was 3.34 pts (“medium-satisfactory”). For food quality, it was taste (3.33 pts), flavor (3.30), temperature (3.34), and nutrition (3.72). Amount of steamed rice (3.35), side dishes (3.22), and dessert (3.25). For menu composition, it was of various kinds of food (3.35), number of side dishes (3.43), and the use of seasonal food (3.50). For food hygiene (3.33) and reflection of students’ opinions (3.03). The biggest unsatisfactory factor was “bland food” (54.05%), and “enhancement of taste” (49.08%) was the one that most generated complaints against school meals. The soup was the type of food most likely to have leftovers (61.92%), and the most cited reason for leftovers was “unfavorable menus” (33.42%). Students were more likely to try unfamiliar foods and foods they previously disliked (28.87%), depending on the consumption of school meals.
Santos et al. (2008) Brazil [61]	To evaluate the protein and caloric content and the acceptability of school meals offered in a state school of elementary education in Porto Velho, Rondônia	SS: *n* = 1 TS: Elementary school P: Students (*n* = 65)	Semi-structured questionnaire Questions (*n* = 6) (1. “Do you usually eat the lunch offered at school?”; 2. “Do you like the lunch served?”; 3. “Do you often repeat your lunch?”; 4. “Do you eat everything?” 5. “Do you eat breakfast before coming to school?”) With answer options (“yes” or “no”) 6. “What lunch would you like to have at school?”	Acceptability	The acceptability of the evaluated school lunch was relatively high. It was found that 81.5% consume and 75.4% like the school lunch served, and 63% do not usually repeat it. Regarding eating everything, 73.8% consume all their school lunches, and 4.6% consume breakfast before school. It is noticed that students do not recognize healthy eating, given the preference for fast food, with 44.6% preferring hot dogs, 30.8% pizza, and 24.6% wanting fruit salad in school lunches.
Danelon et al. (2008) Brazil [62]	To identify children and adolescents’ food preferences in the school environment, analyzing the influence of the coexistence of PNAE and cafeterias on the students’ food behavior	SS: *n* = 6 TS: Elementary, middle P: Students (*n* = 324)	Semi-open form Nº of days/week (1–3 or 4–5) (“likes all preparations” or “dislikes some preparations”) Students selected (among 12 pre-established options) 5 meals that made up an SFP menu	Adherence frequency Acceptance Opinion on the menu preparations Six categories: lunch, juice snack, soup, single dish, sweet, and milk snack	The results revealed the average adherence to the PNAE was 75%. However, only 38.3% participated systematically (4 to 5 days/week). As for acceptance, 66.3% did not like some menu preparations, of which the majority (57.1%) adhered sporadically (between 1 and 3 days/week). The most rejected preparations were the “single dish” type, savory lunch, and soup types (47.2, 32.3, and 21.1%). Among the twelve pre-established options, the students indicated they preferred a complete meal, such as lunch (30.3%) or a snack-type meal with juice (27.1%).
Abranches et al. (2009) Brazil [63]	Analyzing the diet adequacy concerning energy, macronutrients, vitamins A and C, iron, and calcium, as well as the acceptance of the meals offered by public and private daycare centers	SS: Public (*n* = 1) TS: Daycare center P: Students (*n* = 53)	3-point hedonic scale “didn’t like it” (1 pt), “indifferent” (2 pts), or “I liked it” (3 pts)	Acceptance	Of the 53 children, 48 (90.6%) were evaluated for lunch acceptance. Of these, 54.2% responded that they liked the lunch. Lunch had an average score of 2.12, being classified between the hedonic terms “liked” and “indifferent”.
Teo et al. (2009) Brazil [15]	To assess the program acceptance and adhesion by schoolchildren, identifying which aspects of the circumstances of food distribution represent potential determinants of its effectiveness	SS: *n* = 21 TS: Elementary school P: Students (*n* = 686)	Questionnaire Frequency of consumption (“not once”, “1x/week”, “2x/week” “3x/week”, “4x/week”, “daily”) 3-point hedonic facial scale (“liked” to “disliked”) Indifference index = % who neither likes nor dislikes/considers regular Rejection rate = % who do not like school meals	Adherence Adherence rate = % that consumes every day Acceptance Only for those who consume between 1 and 5 x/week (*n* = 633) Acceptance index = % who like school meals	The total daily adherence to school meals was low (23.2%), referring to students who consume them daily, whereas 7.7% do not consume them any day of the week. Adherence was significantly (p < 0.05) higher in municipal schools (29.5%) than in state schools (16.5%). Acceptance was low: 70.8% considered them “good” and 2.4% “bad”.
Bleil et al. (2009) Brazil [64]	To identify the students’ adhesion to the school lunch program as well as the aspects that determine its consumption	SS: *n* = 4 TS: Middle school P: Students (*n* = 167)	Effective attendance index (IAE) % IAE = total students served/total students present at the school × 100 “Effective” adhesion to the program: consumption 4 to 5 times/week Affective preference test 5-point hedonic facial scale (“hated” to “loved’) “Adequate”, “inadequate”, or “did not respond”	Adhesion to the school feeding program High (above 70%), medium (50 to 70%), low (30 to 50%), very low (less than 30%), and frequency of consumption of school meals Acceptability (food preferences) Student opinion (temperature and quantity)	The results showed a high adherence to the program (77%). Of these, 57% consumed school meals 4 to 5 weekly. No significant association was identified between family income, maternal education, nutritional status, and adherence to the program. It was observed that some options offered on the school menu are less acceptable: sweet preparations (41.9%), single dish type (33.2%), and soups (27.1%). The amount was considered adequate by 80.8% of the students. However, the temperature was adequate for only 40.5%.
Caporale et al. (2009) Italy [65]	To examine the association between hedonic responses to school lunch items and the consumption of school lunch among 4–5 years old children, using a selection of twenty menus	SS: *n* = 1 TS: Preschool P: Students (*n* = 71) and parents (*n* = 71)	7-point hedonic scale (“super bad” = 1 to ”super good” = 7) Geometrical mean of the hedonic responses to the entire meal: first and second course, and vegetable Percentage of the amount uneaten of the entire school lunch, relative to the amount served Portion size: weight of 10 standard portions per day (variation of +10% around the mean) 7-point verbally anchored hedonic scale (“dislike very much = 1 to like very much = 7) About 34 school lunch foods	Hedonic ratings Hedonic response index (HRI) Waste index (WI) % of the uneaten food (for each child and dish) Food preference questionnaire (parents indicated hedonic responses of their child)	For students, the second courses were the most preferred (mainly meat or fish), with the highest average score for chicken cutlet and cod sticks (6.6) and the lowest for cod with tomato (5.3). The least preferred were vegetables, higher for potato puree (5.2) and lower for gratin zucchini (3.0). For parents, regarding their children’s preferences, the highest score was for the pasta with tomato and beef stew (5.9) and the lowest for the pasta with beans, gratin cod, and gratin zucchini (4.3). The hedonic response index (HRI) was highly correlated with the waste rate (hedonic responses correlated with the % of the amount not consumed). Thus, when HRI is <5, the waste index is, in all cases, >30.
Song and Moon (2010) South Korea [66]	To investigate the degree of satisfaction with the school lunch program by food service location and examine the sanitary environment by measuring total bacteria in the dining room and classrooms	SS: *n* = 1 TS: Middle school P: Students (*n* = 214)	5-point hedonic scale “least satisfied” (1 pt) to “much satisfied” (5 pts)	Degree of satisfaction Temperature, quantity, taste, quality, color, appearance, and variety of menu	The average student satisfaction was higher in dining room service (2.91) than in classroom service (2.59). With an average satisfaction score for temperature (3.02; 2.68), quantity (3.20; 2.87), flavor (2.81; 2.52), quality (2.47; 2.31), color (2.89; 2.50), appearance (2.92; 2.51), and variety of menu (2.77; 2.46), respectively.
Lazor et al. (2010) USA [67]	To assess the acceptance of soy-based foods substituted for popular lunch items among adolescents in a large urban area with culturally diverse populations	SS: *n* = 5 TS: High school P: Students (*n* = 3.993)	Average serving weight (standard) = average weight of 5 samples = comparison of the weight of the remaining foods with the weight of the standard serving of the item = amount of food consumed compared to what was served Ranges from 0 (nothing of the product consumed) to 1 (the entire product consumed).	Acceptance Formal plate waste study Estimated amount consumed The proportion of product consumed	The estimated average amount consumed (oz) for each popular food was chicken salad (1.70), beef patty (1.81), cooked pasta (5.67), and chicken nuggets (3.98). Regarding the proportion of the product consumed (oz), it was found to be chicken salad (0.92), beef patty (0.92), cooked pasta (0.79), and chicken nuggets (0.98).
Pegolo and Da Silva (2010) Brazil [68]	To evaluate the consumption of energy and nutrients and adherence to the National School Feeding Program (PNAE) by schoolchildren aged between 7 and 14 years	SS: *n* = 6 TS: Elementary, middle P: Students (*n* = 150)	Questionnaire (“yes” or “no”) (“none day”, “1x/week”, “2x/week”, “3x/week”, “4x/week”, or “5x/week”) (“like” or “dislike”)	Adhesion Weekly frequency of consumption (adhesion) Acceptance	Regarding total adherence, 81.3% of students reported consuming meals when separated by the school (1 to 6): 57.8, 91.7, 100, 80.9, 91.7, and 94.4%, respectively. About half (52%) of students from all schools reported frequency of adherence considered effective (four to five times a week). By school, 17.7, 66.7, 91.7, 42.9, 91.7, and 63.9%. As for the opinion of schoolchildren about food, 66.4% of the students from all schools declared that they did not appreciate the preparations and/or food offered. By school, we obtained: 57.7, 63.6, 50.0, 70.6, 45.5, and 85.3%.
Matihara et al. (2010) Brazil [69]	To identify the nutritional value of school lunches and the acceptability of students from a state school in the city of Maringá, PR	SS: *n* = 1 TS: Elementary school P: Students (*n* = 104)	3-point hedonic facial scale (“I really liked the lunch”, “I liked the lunch”, and “I did not like the lunch”)	Acceptability	The results showed that among the 14 preparations analyzed, considering the option “I really liked the lunch”, the one that obtained the greatest acceptance was bread with hamburger and milk drink (87%). The lowest acceptance was rice, beans, and ground beef + dwarf banana (39%).
Lee and Park (2010) South Korea [70]	To investigate school food service satisfaction and menu preferences of high school students in the Iksan, Cheonbuk area	SS: *n* = 4 TS: High school P: Students (*n* = 692)	Self-administered questionnaire 5-point Likert scale (“Very satisfied” = 5 to “very dissatisfied” = 1) (rice, soup, vegetable namul, meat, fish, kimchi, fruit) (“dislike the taste”, “too big portion size”, “stomach ache when eat the food”, “not digest”, “not appetite”, “never having the food”) (“more quantity of food”, “more taste food”, “various recipes”, “balanced nutrients”, “hygiene”) 5-point Likert scale (“like very much” to “dislike very much”)	Satisfaction Menu (taste, smell, quantity of the main dish, quantity of side dish, *nº* of side dish, food color, harmony of food, nutrients, quality, and salty taste) Food temperature (rice, soup stew, jorimpan-fried, kimchisalad), School Meals Improvements Food that leaves leftovers in school meals (%) Reasons for leaving leftovers (%) Items that you want to improve in school meals (%) Menu preference (general menu, main dish, soup and stews, side dishes, fruits, beverage, and teawater)	The average satisfaction score for the menu was 2.8, with the highest score for the quantity of the main dish (3.2) and the lowest for the quantity of the side dish (2.5). As for food temperature, 3.1 were obtained, higher for rice (3.8) and lower for jorimpan-fried (2.9). Both were considered low. The main reason they left the food was “dislike the food taste” (65.3%), and the food with the most leftovers was vegetable namul (41.3%). According to the students, there is a need for improvements for “more taste food” (39.3%), “various recipes” (18.8%), and “more quantity of food” (8.1%). The average score for menu preference was 3.7, with the highest score for fruits (4.2) and the lowest for muchim (3.1).
Chu et al. (2011) USA [22]	To compare the acceptance of whole-grain pancakes and tortillas to refined-grain counterparts when served as part of the school meal	SS: *n* = 10 TS: Elementary, middle, and high school P: Students (*n* = not informed)	% of consumption = weight per serving × *nº* of serving − total plate waste/weight per serving × *nº* of serving × 100% 5-point hedonic facial scales (elementary schools) or 9-point hedonic scales (middle and high schools)	Aggregate plate waste Acceptance Overall liking, taste, color, and softness (higher rating indicates higher acceptance)	The average consumption of refined pancakes and tortillas was 78% and 79%, respectively. For overall liking, in elementary schools, pancakes averaged 4.2, and tortillas averaged 4.0. In middle and high schools, the average pancake score was 6.3, and tortillas 6.8.
Chesser (2013) USA [23]	To determine how middle school student’s participation in the NSLP is influenced by the student and school demographics, school lunch practices and policies, and student attitudes regarding cafeteria setting, food acceptability, and school food service staff	SS: *n* = 27 TS: Middle school P: Students (Phase I: 6 focus groups *n* = 82 and Phase II: *n* = 648)	Phase I (focus group) Questionnaire Questions (*n* = 6) Open-ended questions Phase II Questions (*n* = 10) Likert-type 5 points scale ranging “Strongly disagree” (1 pt) to “strongly agree” (5 pts)	Food acceptability The frequency of consumption, reasons for eating, influences, what you like most and least, and the variety, if they are healthy, suggested changes Temperature, appropriately cooked, way are paired/served, attractive, that I like, quality foods, portion sizes are large enough, tastes good, serves food the way I like them cooked, serves foods like I eat at home	Regarding phase I, the students overall suggested more variety and improving cooking methods and presentation techniques, bigger portion sizes, and changing the types of foods to be more like what is served at home. They commented on the lack of seasoning and flavor. Reported wanting more of a say in selecting the components of their meal. The Likert-type scale responses showed low food acceptability (2.44). Food served at an acceptable temperature (2.92) received the highest score. The cafeteria serving foods the way students like them cooked and serving food as students eat at home scored the lowest (1.97).
Dias et al. (2013) Brazil [71]	To analyze the quality, acceptance, and plate waste generation of the food offered in an education center for youths and adults located in the urban zone of Cuiabá, Mato Grosso, Brazil	SS: Education center for youth and adults (*n* = 1) TS: Teaching youth and adults (EJA) P: Students (*n* = 174)	5-point hedonic verbal scale (“hated” to “loved”) Distributed meal portion (g) = total weight of the preparations produced − total weight of the leftovers of the preparations/*nº* of distributed meals Per capita leftover intake (g) = total leftovers left by students/*nº* of meals consumed Rest intake index = per capita of rest intake (g)/distributed portion (g) × 100	Acceptability index “Accepted” = ≥85% (adding “liked” and “loved”) Rest ingestion index “Accepted” = ≥90%	Meal acceptance obtained higher rates than the recommendations in the morning (86.50%) and afternoon (93.65%) periods. When analyzing the rest intake index, similar rates (7.50%) were observed in both periods, representing acceptance.
Cruz et al. (2013) Brazil [72]	To assess the uptake of school meals offered to students of a municipal school	SS: *n* = 9 TS: Elementary school P: Students (*n* = 990)	5-point hedonic facial scale (“hated” to “loved”)	Acceptance “Not accepted” ≤ pts	All preparations showed an average acceptance above three points. Therefore they were accepted. The most accepted snack was the bowl cake with juice (4.39), and the least accepted was the sweet rice (2.99). When asked about the foods they would like to be served by the school. Of the 206 students who responded, they included hot dogs (24.3%), cookies (22.8%), soft drinks (13.6%), and stuffed cake (12.1%).
Barrios et al. (2013) Chile [73]	To determine Kcal provided and consumed from breakfast and lunch trays, respectively, acceptance of the preparations and assess if there was an association between Kcal consumed and nutritional status	SS: *n* = 6 TS: Preschool P: Students (*n* = 199)	The technique of weighing by difference (delivered and consumed) The average weight of each food delivered = averaging from 4 trays selected “Acceptability” variable for each preparation = grams administered − grams consumed × 100 to obtain a percentage. Asked daily: “liked” or “disliked” each preparation separately	Acceptance Real intake of food	A total of 148 breakfast trays and 460 lunch trays were delivered, and the actual consumption came from measurements performed on 429 and 1491 trays, respectively. As for the proportion of positive responses (I liked) about lunch, for salad, only in 44.1% of the trays the children responded that they liked it. The acceptance of main dishes was very good (86.2%), with legumes being the most accepted (over 90%). There were 67.1% acceptance for desserts, the highest for natural fruits and jellies, and the lowest for dairy desserts. As far as breakfast is concerned, dairy products are mostly well-accepted. The “liked” aspect presents a good correlation with the acceptability aspect; the least consumed preparations correspond to those with the highest proportion of “did not like”.
Leme et al. (2013) Brazil [12]	To identify and justify adolescents’ food choices during recess at school and to get to know the school staff’s vision about the student’s acceptance of the Brazilian School Meal Program	SS: *n* = 1 TS: Middle school P: Students (*n* = 83) and cooks (*n* = 4)	Questionnaire Open question: “During class breaks, do you usually eat and/or drink something?” (“yes” or “no” and “why?”) Interview Speech questions (*n* = 2) (“What is your perception of students’ opinion about school feeding?”) and (“In your opinion, should something be changed in the food offered by the school feeding program to students? Why?”) Analysis of the questionnaire and interview carried out = collective statements (written in the 1st person singular) made with extracts from different individual statements	Acceptance Employees’ perception (the questions were recorded and transcribed into a database) Qualitative methodology of the collective subject discourse (CSD)	Adolescents prefer “competitive” foods sold in establishments close to the school and/or brought from home and do not like the meals offered by the program. They adhere to it, as it is the only alternative they have at school. In the view of employees, 63.4% believe that teenagers like school lunches. However, they considered that some of the foods are not part of the adolescents’ eating habits and that some foods do not attract them, which is why they waste them. A total of 54.6% of employees agree with changes in the menu and suggest changes related to food composition and its flavor and texture characteristics.
Silva et al. (2013) Brazil [74]	To analyze the Brazilian School Nutrition Program from the standpoint of students attending state schools in Minas Gerais	SS: *n* = not informed TS: Elementary, middle, and high school and Teaching youth and adults (EJA) P: Students (*n* = 1.500)	Semi-structured questionnaire (“sometimes”, “always”, or “does not consume”) (“excellent”, “very good”, “good”, “fair”, or “poor”) Those who considered it “regular” or “poor”, reported the reasons % of students who consume school meals (“no”, “sometimes”, or “always”) Frequency (%) of food appearance/preparations in school menus (with 12 items) (“does not appear”, “1x/week” to “5x/week”) Frequency (%) of improvement suggestions suggested by students (1 or more suggestions)	Food consumption School food quality (acceptance and adhesion) Acceptance “Effective” = “excellent” or “very good” Adhesion “Effective” = consumption ≥4x/week Improvements to school meals	As for school meals, 44.8% reported consuming them “sometimes” and 47.7% “always”. Effective acceptance to the program was 28.8% (“excellent and “very good”), with no significant difference between the levels of education. Among those who considered it “regular” or “poor”, one of the main reasons was the “monotony of the menu”, by repetition of “noodles” or “soup”. Effective adherence was 45.1% (≥4x/week) and was significantly higher among EJA students (72.9%) when compared to EJA students. High school (44.2%) and elementary school (41.2%). In total, 73.5% of the students suggested improvements in school meals, the most frequent being “include fruits in the menu” (27.2%) and “change the menu” (24.5%).
Yang et al. (2013) South Korea [75]	To analyze the quality attributes, quality factors, and customer satisfaction in school food service and to provide suggestions for improving the school foodservice environment	SS: *n* = 96 TS: Elementary, middle, and high school P: Students (*n* = 5.768), parents (*n* = 2.044) and faculty (*n* = 1.978)	Questionnaire from the School Meal Satisfaction Survey 2009 (MEST2009) (partially modified and supplemented) 5-point scale Results = presented by converting them to a perfect score of 100	Foodservice quality Quality attributes (taste, proper temperature, adequate quantity, menu variety, nutritional foods, food sanitation, and quality of food ingredients)	As for food service quality, the average scores of students, parents, and faculty were 76.5, 79.9, and 89.0 pts, respectively. The highest scoring attributes were students (nutritional food 79.4, temperature and quality of food ingredients 77.3), parents (nutritional food 82.2, food sanitation and quality of food ingredients 81.0), and faculty (food sanitation 92.0 and quality of food ingredients 90.7). The lowest were students and parents (quantity 73.0 and 77.4) and faculty (menu variety 87.2). When evaluated by place of distribution of meals (classroom or dining hall), for students, it was 77.7 (*n* = 1.401) and 76.1 pts (*n* = 4.369). For parents, it was 80.2 pts (*n* = 484) and 79.8 (*n* = 1.561), and for faculty, 87.6 (*n* = 462) and 89.5 (*n* = 1.518), respectively.
Turconi et al. (2013) Italy [76]	To determine the acceptability, waste, and nutritional adequacy of lunches served in all public primary school canteens in Pavia, Northern Italy	SS: *n* = 13 TS: Elementary school P: Students (*n* = 448)	4-point scale (“all”, “half”, “none”, “second helping”) The nutritionist asked the child about the reason for not consuming (free answer)	Acceptability Visual estimation of food consumption	Of the 448 children, 415 had lunch in the school canteen, of which 32, 55, and 328 had lunch for one, two, or three days, respectively. Over the three days, the total number of observations was 1126. Only 49.6% fully consumed the first course, the main course (35.4%), vegetables (20.9%), and desserts (54.1%). The most refused were vegetables (69.2%). Portion sizes were often too big for children, averaging 75 g (excluding soups, vegetables, and broths). In the case of non-consumption, they usually answered: “I don’t like it”, “Mom cooks better”, “I like it, but I’m not hungry”, or “It’s too cold”.
Rodriguez-Tadeo et al. (2014) Spain [24]	To assess the acceptance of food by weighing food leftovers and validation of a methodology for visual estimation in school canteens of Murcia	SS: *n* = 11 TS: Elementary school P: Students (*n* = not informed)	Estimate of leftovers (weight of the leftovers of each food and calculation of the net weight consumed) Categorical scale: 1 (0–25%), 2 (26–50%), 3 (51–75%), 4 (76–100%)	Acceptance Accepted: 75% of the portion consumed Visual estimation	The dishes with the highest proportion of leftovers were the main dishes based on vegetables, such as purees and salads, pasta, and rice (cold line) and pulses, salads and stews with fish (hotline), and the second dishes based on vegetables, poultry and fish. fruits (desserts) and bread, especially wholemeal. The visual scale is a viable tool to measure acceptance indirectly.
Angeles-Agdeppa et al. (2014) Philippines [77]	To investigate dietary intakes and acceptance of nutritionally balanced school meals (“nutri-meals”) as compared with regular (“baseline”) school meals among Filipino students	SS: *n* = 1 TS: High school P: Students (*n* = 112)	7-point hedonic scale (“like very much” to “dislike very much”)	Acceptability Overall liking, taste, and appearance	As for overall liking, the vast majority of students (99%) liked the baseline meals, attributing “like very much”, “like moderately”, or “like slightly”. Mean scores for taste and appearance differed significantly between baseline meals and nutri-meals.
Valeriani and Sturion (2014) Brazil [78]	To check the acceptance and adhesion rates in the management model schooled	SS: *n* = 51 TS: Elementary, middle, and high school P: Students (*n* = 35.379)	Effective attendance index (IAE) = total number of students effectively served/total number of students enrolled × 100 % of acceptance = count of the *nº* of dishes with leftovers and totaled according to the percentage of leftovers (0%, 25%, 50%, 75%, 100%) Average acceptance % = 100 − [(T0 × 0) + (T25 × 25) + (T50 × 50) + (T75 × 75) + (T100 × 100)]/T0 + T25 + T50 + T75 + T100	Adherence Students enrolled/present Very low (≤33%, ≤40%), low (34 to 51%, 31 to 58%), medium (52 to 67%, 59 to 80%), and high (≥68%, ≥81%) Visual estimate of leftovers in each plate Acceptance “Accepted” ≥85%	The average adherence was 49% (enrolled) and 56% (present), classified as low. There was a difference at a 5% significance level between adherence in the morning and the afternoon. The presence of a commercial establishment, the period (morning and afternoon), and the menu type influenced adherence. From the “Visual Estimate of Leftovers in each Plate” performed in 69% of the meals served, an average acceptance rate of 87% was obtained, indicating good acceptance of the preparations.
De Oliveira et al. (2015) Brazil [19]	To examine the acceptability of school lunches among public elementary school children and determine factors influencing school meal acceptance	SS: *n* = 1 TS: Elementary school P: Students (*n* = 189)	5-point face hedonic scale (“hated”, “disliked”, “neither liked nor disliked”, “liked”, “loved”) Residual index (RI) = rejected meal weight (RMW)/served meal weight (SMW) × 100 ARI = 100 − RI SMAI (%) = *nº* of students in the 2nd and 4th grades that had lunch at school/*nº* of students in the 2nd and 4th grades who attended school × 100 MRI (%) = *nº* of students on the 2nd and 4th grades that repeated lunch/*nº* of students on 2nd and 4th grades that had lunch at school × 100	Acceptance Face hedonic scales (FHS) “Accepted” ≥ 85% Residual index method (RI) RI ≤ 10% “adequate” ARI > 90% “accepted” School meals adherence index (SMAI) High (>70%); medium (50 ≤ SMAI ≥ 70%); low (30 ≤ SMAI ≥ 50%); very low (SMAI < 30%) Meal repetition index (MRI) (any item on the menus)	Results have shown that, on average, menus were not accepted. The face hedonic scales (FSH) results revealed that seven menus had an acceptance percentage greater than 85%. The pasta was the dish children “liked most” (26.4%), and the salad was the “most disliked” one (31.2%). For the residual index method (RI), no menu was accepted. The menu that showed the greatest acceptance (ARI = 86.6% or RI = 13.2%) was chicken and pasta with tomato sauce and black beans. The school meals adherence index was 64.5% (medium). For the meal repetition index (MRI), three menus showed the highest values (48.0%), of which two menus included grounded meat and the other mixed dish with pasta and chicken as the main course.
Carlini et al. (2015) Brazil [79]	To know the acceptability and the adhesion level of the school feeding offered to the high school students of this institute	SS: Federal Institute of Sertão Pernambucano, Salgueiro campus (*n* = 1) TS: High school P: Students (*n* = 56)	AI = *nº* of students who consumed the meal/*nº* of students present at school × 100 Questionnaire Questions (*n* = 3) Do you usually eat (“yes” or “no”), days a week (“1 day/week” to “5 days/week”), do you like the food offered (“yes, all”, “no, some“, or “I don’t like any”) Questionnaire Questions (*n* = 2) (“always good”, “sometimes good” or “never good”; “a lot”, “good”, or “a little”; “yes” or “no”), respectively)	Adherence index High (>70%), medium (50 to 70%), low (30 to 50%), and very low (<30%) Adherence and acceptance Temperature and quantity	There was a high rate of adherence (74.43%). As for acceptance, 61.82% of students said they liked all the preparations offered, and 38.18% did not like some preparations. The temperature of the meals was always good for 62.50%. However, 35.71% reported that sometimes some were cold or very hot. The amount was classified as good by 60.71% and insufficient by 39.29% of the students. 62.07% responded that the place was uncomfortable because there was no place for everyone to sit.
Smith (2015) USA [80]	Evaluate the food choices and consumption patterns of elementary and middle school students participating in the National School Lunch Program (NSLP) and compare students’ average nutrient intake from lunch to NSLP standards (Chapter 3)	SS: *n* = 5 TS: Elementary, middle P: Students (*n* = 899)	Digital photography (reference photographs and post-consumption of each student’s tray) The average weight of the 5 portions for each food item = standard when estimating the weight of food consumed % of food wasted = weight of each remaining (uneaten) food item/average weight of the 5 reference portions	Visual plate waste estimation Plate waste	As for food choices, all students chose an entrée. For canned and fresh fruits, 59% and 56% (ES) and 52% and 39% (MS), respectively. Regarding vegetables: 56% (ES) and 34% (MS). Approximately 96% (ES) and 82% (MS) selected milk with lunch, of which three-quarters were fat-free chocolate. As for the percentage of each menu item wasted, for ES and MS: entrée (23.8%; 19.2%), canned fruit (37.3%; 37.6%), fresh fruit (37.0%; 47.4%), vegetable (33.6%; 30.6%), grain (44.6%; 20.0%), and milk (32.6%; 21.2%).
Smith (2015) USA [80]	To determine middle school students’ satisfaction with the school lunch experience, using two validated surveys; the Middle/Junior High School Student Participation Survey and the Middle/Junior High School Student Non-Participation Survey, both developed by the National Food Service Management Institute (NFSMI) (Chapter 4)	SS: *n* = 3 TS: Middle school P: Students (*n* = 473)	Section I 5-point Likert scale (1 = ”strongly disagree” to 5 = ”strongly agree”) for participation (When I eat school lunch…) and non-participation (My reason for not eating school lunch is…) Participation Survey (consumption >3 days/wk) Section II Selection of their top 5 factors (14 provided) why they eat school lunch Selection of top 5 factors (14 listed) that would encourage them to eat school lunch more often	Food preference Menu offers healthy choices, variety, properly cooked food I like, taste, fresh, satisfaction after eating, smell, quality, looks appealing, tastes homemade “Agreement” = 3 pts	As for the participation survey (*n* = 288 students), the statement with the highest degree of agreement was “The menu offers healthy choices” (average of 3.76, 64.9%), and the one with the lowest level was “The food tastes homemade” (2.34, 21%). Regarding the non-satisfaction survey (*n* = 185 students), the statement with the highest degree of agreement was “The food does not look appealing” (3.78, 67.1%), and the one with the lowest level was “There is no variety of food choices” (3.06, 33.6%). In Section II of the Participation Survey, the top reason for eating school lunch was “I am hungry” (77%, *n* = 222), and the least cited reason was “I get a homemade meal” (2%, *n* = 6). In Section II of the Non-Participation Survey, more than 60% said they would be more likely to eat school lunches with better-tasting food, quality, and shorter lines.
Tuorila et al. (2015) Finland [81]	To identify factors affecting the acceptance of school meals	SS: *n* = 2 TS: Elementary, middle P: Students (*n* = 127)	Form (questions = 3) (“regular” or “vegetarian”; or “did not eat”; 1 = “not at all” to 7 = “very hungry” or ”can’t say”; “elated”, “angry”, “sad”, “pleased”, “disappointed”, “happy”, “nothing”; 1 = “really bad” to 7 = “really good”) 7-point Likert scale (1 = really bad to 7 = really good) 7-point hedonic scale (1 = “really bad” to 7 = “really good” or ”did not eat”) 7-point hedonic scale (1 = “far too cold”, 4 = “just right, 7 = ”far too hot”; 1 = “far too little spices”, 4 = “just right”, 7 = “far too much of spices”; 1 = “far too little salt”, 4 = “just right”, 7 = “far too much salt”) (“milk”, “sour milk”, “water”, ”nothing’) Open-ended voluntary question (Why a food was good or bad?) 7-point hedonic scale (1 = ”really bad” to 7 = “really good”)	Which meal had selected Perceived hunger prior to eating Emotions when first seeing the food of the day Overall meal experience Meal acceptance Hedonic ratings Entire meal (main dish, salad, bread) Main dish (temperature, spiciness, and saltiness) Chosen beverage General attitude to school food (appearance and taste), bread, salad	The average hedonic ratings for the entire meal were 5.1 (3rd grade), 3.9 (6th grade), and 4.0 (8th grade). For the main course (5.1, 3.9, and 4.0), salad (5.0, 4.2, and 3.9), and bread (6.1, 4.7, and 4.6), respectively. As for the main dishes, it was obtained for temperature (3.9, 3.5, and 3.5 pts), spiciness (3.8, 3.3, and 3.3 pts), and saltiness (3.8, 3.4, and 3.5 pts). They used hedonic terms for why a meal was good or bad (open question) (good/bad; like/dislike). They typically gave descriptions when food was not much appreciated (“why bad?”), referring to perceived defects in appearance (vague; slimy), texture (lumpy; mushy; sticky), or taste (bland; spicy).
Ali and Akbar (2015) India [16]	To analyze the difference in students’ preferences on the weekly menu of the school mid-day meal (MDM) program in Uttar Pradesh, India	SS: *n* = 480 TS: Elementary, middle P: Students (*n* = 2.400)	Questionnaire Questions (*n* = 24) Dichotomous and Likert scale % of students that reported eating school mid-day meal % that reported no deviation in day-wise menu % that reported 1st preference on day-wise menu % that reported whether they want to have a change in the existing weekly meal menu	Eating behaviors (quantity and quality of meals, serving of meals, preferences on weekly menu, and want to have a change to the existing weekly meal menu)	More than 90% of students eat MDM in the school per the weekly menu, and the same percentage reported that there was no deviation from the approved menu as prescribed by the MDM authority, Lucknow, for each day. According to the χ2-test, the students’ choices on the school meal menu differ significantly across weekdays. Rice pulses or rice sambar served on Tuesday is reported to be the first preferred food (29,9%), and the Indian bread pulses or Indian bread-vegetables or daliya (Thursday) is the least preferred (4%). There was a significant difference in weekly menu choices by gender, kitchen types, rural and urban locations, and geographical regions. About 27,2% reported that they wanted to have a change in the menu, and the most desired was puri-vegetables.
Basaglia et al. (2015) Brazil [8]	To evaluate the acceptance of school lunches in five state schools in the city of Amparo, SP, through acceptance tests by facial and verbal hedonic scales	SS: *n* = 5 TS: Elementary, middle, and high school P: Students (*n* = 135)	5-point hedonic facial scale (2nd to 5th grade) or 5-point hedonic verbal scale (6th to 3th grade of high school) (“hated” to “loved”)	Acceptance “Accepted” = ≥85% (Adding “liked” and “loved”)	The acceptance by students, adding the options “I liked” and “I loved it”, was 83.92% (2nd to 5th year) and 74.14% (6th to 3rd year of high school). Those who reported disliking (adding “I hated” and “I didn’t like”) were 7% and 14%, respectively. Thus, the acceptance of the first group was higher than that of the second. As for acceptability, it was considered good for both groups. However, they did not reach the recommended percentage to be considered accepted.
Ferreira et al. (2015) Brazil [82]	To evaluate some variables involving food offered by the School Nutrition National Program in those municipal schools in Palmas, TO	SS: *n* = 25 TS: Elementary, middle P: Students (*n* = 875)	Questionnaire Questions (*n* = 4) Consumption of school meals (“yes” or “no”) Consumption frequency (“no day”, “1x/week” to “5x/week”) Likes the preparations offered (“all”, “some”, “does not like any”, or “does not consume”) Amount of food offered (“exaggerated”, “sufficient”, “insufficient”, or “does not consume”) 5-point hedonic facial scale (“hates”, “dislikes”, “partially likes”, “likes”, or “likes very much”)	Acceptability “Satisfactory”: ≥85% Degree of satisfaction	A total of 91.89% of the students consumed the food offered at school. Of these, 52.91% consumed daily, and 8.11% did not. Only 33.71 liked all the dishes served, and 1.95% did not like them. For the majority (62.06%), the amount was sufficient. As for the degree of satisfaction, 27.66 “likes it”, 25.94% “likes it very much”, and 31.71% “likes it moderately”, totaling 85.31%. 3.77% did not like it, and 0.80% hated it. Thus, the acceptability of the food offered was considered satisfactory.
Smith et al. (2015) USA [83]	To determine the satisfaction of high school students with the school lunch experience, using two validated surveys: (i) the Middle/Junior High School Student Participation Survey and (ii) the Middle/Junior High School Student Non-Participation Survey. The two surveys were developed by the National Food Service Management Institute (NFSMI)	SS: *n* = 3 TS: Middle school P: Students (*n* = 473)	5-point Likert scale (1 = strongly disagree to 5 = strongly agree) Participation Survey (3 or more days/week) When I eat school lunch… Non-Participation Survey (fewer than 3 days/week) My reason for not eating school lunch is… (answer options written in the negative form) Participation Survey: selection of the top 5 factors (14 provided) why they eat school lunch Non-Participation Survey: selection of the top 5 factors that would encourage them to eat school lunch more often	Satisfaction Section 1 Food preference factor (*n* = 11 statements) (menu offers healthy choices, variety, adequately cooked, has food I like, tastes good, fresh, satisfied after I eat, smells good, quality of the food is good, food looks appealing, food tastes homemade) Section 2	As for the participation survey (*n* = 288 students), the total average was 3.07, the statement with the highest degree of agreement was “The menu offers healthy choices” (3.76, 64.9%), and the one with the lowest level was “The food tastes homemade” (2.34, 21%). For the non-satisfaction survey (*n* = 185 students), the total average was 3.07, the statement with the highest degree of agreement was “The food does not look appealing” (3.782, 67.1%), and the one with the lowest level was “There is no variety of food choices” (3.06, 33.6%). In Section II of the Participation Survey, the top reason for eating school lunch was “I am hungry” (77%, *n* = 222), and the least cited reason was “I get a homemade meal” (2%, *n* = 6). In Section II of the Non-Participation Survey, more than 60% said they would be more likely to eat school lunches with better-tasting food, quality, and shorter lines.
Silva et al. (2016) Brazil [84]	To evaluate food waste through the acceptance and the school feeding membership served in three public schools of elementary school II of the municipal school system, located in the city of Itapetinga, BA	SS: *n* = 3 TS: Middle school P: Students (*n* = 720)	5-point hedonic scale (“disliked a lot” to “liked a lot”) Acceptance rate (%) = 100 − % of rejection 3-point hedonic scale (“slightly seasoned/very cold/a little”, “ideal”, “highly seasoned/very lot/a lot”) Questionnaire Questions (*n* = 4) (“yes”, “no”, “no answer”)	Acceptance (for each preparation served) Acceptability rate (seasoning, temperature, and quantity served) Adherence and degree of satisfaction (if you eat, like it, usually repeat it and eat before going to school)	The school lunch acceptability index was below 85% (a limit established by Brazilian legislation). That is, it was not well accepted by students based on their preferences. Most responded that the temperature (56.91%), seasoning (54.5%), and quantity (58.25%) of the meals served were ideal. Of the three schools, two showed good adherence rates (over 80%), and one had a low rate (57%). Most (68%) liked the preparations served, and the main reasons for not consuming were: preferring to buy food in the canteen, not liking the meals served, the meal having little seasoning, or not feeling hungry in the morning. Regarding satisfaction, 23.3% have the habit of repeating their lunch, and 76.3% eat before going to school.
Balestrin et al. (2016) Brazil [6]	Evaluate the acceptance of food in an elementary school	SS: *n* = 1 TS: Elementary, Middle P: Students (*n* = 138)	5-point hedonic facial scale (1st to 5th grade) or 5-point hedonic verbal scale (6th to 8th grade) (“hated” to “loved’)	Acceptability index “Accepted” = ≥85% (adding “liked” and “loved”)	A total of 78.5% of students from 1st to 5th year marked the options “I liked” and “I loved”. A similar result was found among students from the 6th to the 8th grade (72.6%). Thus, they were not accepted, as the percentages of acceptability were not higher than 85%, as the National School Feeding Program recommended.
Silva and Barros (2016) Brazil [85]	To evaluate the acceptability of school meals at the Municipal School of Child Education and Primary Cecilia Estolano Meireles	SS: *n* = 1 TS: Teaching youth and adults (EJA) P: Students (*n* = 20)	Semi-structured questionnaire Questions (*n* = 9) (2 to 9 answer options) Tips to improve school lunch	Acceptability The habit of consuming, consumption frequency, like the offered meals, foods they do not like, reason leading to eating, temperature, quantity, bring the food home, food provided they like best, tips of students to improve school lunches	85% of students have the habit of consuming school meals, and most (70%) consume 5 times/week. Only 15% like all meals and 70% do not like some. As for the foods they do not like, the most cited was bread with butter, sweet biscuit/salty, and tea (by 20% of the students each). The main reason to eat was to feel hungry. The temperature was considered good (55%), and the amount was considered sufficient (85%). Everyone (100%) usually brings food from home; the most they like are hot dogs and juices (30% each). The most reported tip to improve school meals was to diversify the menu.
Maietta and Gorgitano (2016) Italy [86]	To analyze to what extent pupils value the characteristics of the state school food service and identify which variables affect the degree of pupils’ satisfaction with the quality of school meals	SS: *n* = 33 TS: Elementary school P: Students [87] (*n* = 2.210)	Questionnaire Questions (*n* = 2) (“not satisfied” (level 1), “poorly satisfied” (2), “sufficiently” (3), “fully” (4), or I do not know Food characteristics/menu What do you wish for? Which foodstuff do you leave most often?	Satisfaction Level of pleasantness of eating at school Level of school food tastiness Reasons for dissatisfaction Tastier food, a dessert, more meal variety, more fresh fruit and/or vegetables, hotter food, a larger serving Foods that are most often not eaten Pasta, fish, vegetables, bread, fruit, meat	Regarding the taste level of the food, 48% said they were sufficiently or completely satisfied, and 2% could not. The main reason for dissatisfaction was the absence of tasty food. As for the level of pleasure in eating at school, 53% of students are sufficiently or completely satisfied, and 1% could not say. The foods most often not consumed are pasta, fish, and vegetables. When controlling for variables (characteristics of the student, family, school, food service, and catering company), it was observed: (i) the size of the catering company negatively impacts student satisfaction with the food service, and (ii) the estimated average cost of production of the meal is positively associated with student satisfaction.
Bez (2017) Brazil [87]	To evaluate the school feeding menus regarding the acceptance and compliance of the nutritional parameters established by the PNAE in a municipal school in the city of Francisco Beltrão, PR	SS: *n* = 1 TS: Elementary school P: Students (*n* = 150)	Reject percentage = rejected meal weight × 100/distributed meal weight % of acceptance = 100 − % of rejection	Rest ingestion index “Accepted” ≥ 90%	Of the five days of evaluation, in three, there was a percentage of acceptance greater than 90% within the program’s parameters, meaning that the menus offered were accepted. However, on the other days, an index of 89.53% and 78.27% was obtained, being considered not accepted by the students.
Sanabria et al. (2017) Paraguay [14]	To evaluate the degree of acceptance and percentage of nutritional requirements adequacy of the scholar lunch of children from two public schools from Asunción	SS: *n* = 2 TS: Elementary, middle P: Students (*n* = 102)	5-point hedonic facial scale (“I don’t like it at all” to “I love it”) Percentage of food consumption = final weight/initial weight × 100	Degree of acceptance Food consumption	The degree of acceptance of school lunch was conditioned by the type of menu offered on the day. As for the facial hedonic scale, about 3 out of 10 children said the food was to their liking. When estimating the percentage of consumption, more than half consumed about 75% or more of the daily meal. However, when the menu included legumes, the acceptance percentage for both indicators was lower. The majority (80%) stated that the amount was sufficient and satisfied them. Already 8 out of 10 said they wanted to add one more meal to the daily menu, and ¼ chose Milanese as their favorite meal.
Da Silva et al. (2017) Brazil [88]	To assess whether specific food education activities in public schools could improve food knowledge and promote the acceptance of meals planned by the National School Feeding Program	SS: *n* = 3 TS: Elementary school P: Students (*n* = 243)	5-point hedonic facial scale (“hated” to “loved’) and Questionnaire Questions (*n* = 2) (What did you like the most/least about the preparation?) Distributed meal portion (g) = total weight of the preparations produced − total weight of the leftovers of the preparations/*nº* of distributed meals Per capita leftover intake (g) = total leftovers left by students/*nº* of meals consumed Rest Intake index = per capita of rest intake (g)/distributed portion (g) × 100 Questionnaire Questions (*n* = 4) Accept (yes or no); *nº* of days/wk accepted (1, 2 to 3, 4 to 5); consumes school meals and buys food (yes or no); *nº* of days/wk you buy food (1, 2 to 3, 4 to 5)	Acceptance “Accepted” = ≥85% (Adding “liked” and “loved”) Rest ingestion index “Accepted” = ≥90% Adherence and acceptance	Most students (81%) accept school meals. Of these, 46% consume 4 to 5 times a week. The information obtained through the hedonic scale and the adherence frequency questionnaire showed that the most accepted foods were rice and beans (both 57%), and the least accepted was steak (5%). The most rejected food was beet (28%), and the least rejected was sweet potato (3%).
Carvalho et al. (2017) Brazil [9]	Identifying adherence to, and acceptance of school feeding, and analyzing the factors associated with non-adherence/non-acceptance in full-time public schools in Goiânia, Goiás, Brazil	SS: *n* = 20 TS: Elementary, middle P: Students (*n* = 359)	5-point facial hedonic scale (“love” to “hate”) Questions: objective (*n* = 2) (“adequate”, “inadequate”/“much”, “good/sufficient”, “little”)	Acceptance “Accepted” = ≥85% (adding “liked” and “loved”) Temperature and amount of food Adherence “Adherence” = consumption of each meal 4 to 5 days/wk High (>70%), medium (50 to 70%), low (30 to 50%), and very low (<30%)	As for acceptance, none of the meals reached the minimum acceptance of 85%, where: morning snack (54%), lunch (72%), and afternoon snack (65%). Food temperature was considered adequate for 89.5%, 88.8%, and 87.9%, and the amount was “good/sufficient” for 54.7%, 57%, and 63.4% of students, respectively. For adherence, it was high for lunch (95%) and afternoon snacks (78.0%) and low for morning snacks (44%). Factors associated with non-adherence were: (i) the presence of > four people in a household, (ii) having meals in a refectory, (iii) the meal location being considered uncomfortable, and (iv) a negative evaluation of utensils used in eating meals. Factors associated with non-acceptance were age >10 years, female sex, the negative evaluation of utensils used in meals, and inadequate food temperature.
Pedraza et al. (2017) Brazil [89]	To characterize the National School Feeding Program (Programa Nacional de Alimentação Escolar—PNAE) in public schools, considering structural and procedural aspects and the acceptance of school meals	SS: *n* = 17 TS: Elementary school P: Students (*n* = 1.081)	Questionnaire Questions (*n* = 5) (“good”, “fair”, or “poor”), (“yes” or “no”), (open-ended question), (“yes”, “no”, or “sometimes”), and (open-ended question), respectively	Acceptance (perception, daily frequency of consumption, foods with the highest rejection, habit of taking money to school and the food bought with it)	The results related to acceptance of school feeding showed that 75.02% (*n* = 811) of the students considered it to be good, 23.13% (*n* = 250) considered it regular, and 1.85% (*n* = 20) it was not good. 36.26% (*n* = 392) of the schoolchildren reported not eating school meals daily. Soup (27.57%, *n* = 298) and milk rice (11.29%, *n* = 122) were the most cited items when asking the schoolchildren about the foods they disliked. The percentage of students who reported taking money to school was 57.50% (*n* = 622), of which 8.29% (*n* = 52) indicated always doing so, while 49.21% (*n* = 532) took it at times. Of the children who claimed to take money to school, 45.41% (*n* = 282) reported spending it on popcorn and 7.25% (*n* = 45) on sweets or candies, which are the most mentioned options.
Raphaelli et al. (2017) Brazil [90]	To evaluate the adhesion and acceptability of school meals menus in a rural municipality, specifically, that of Barão do Triunfo, RS, Brazil	SS: *n* = 2 TS: Preschool, elementary, middle P: Students (*n* = 240)	AI = *nº* of students who consumed the meal/*nº* of students present at school × 100 5-point hedonic facial and verbal scale (“liked extremely”, “liked moderately”, “neither liked/disliked”, “disliked moderately”, or “disliked extremely”) AI = sum of votes from the cards “I liked it extremely” and “I liked it moderately”/*nº* of students who had the meal × 100	Adherence index “Good adhesion” = at least 85% Acceptance Acceptance index “Good acceptance” = at least 85%	The evaluation of acceptance by the hedonic scale showed that 56.32% and 22.42% of the students liked the school lunch extremely and moderately, respectively. By school, the smallest showed that 82% of the votes in the scale were for the option “I liked it extremely” and 10% for the option “I liked it moderately” of the menus. In the larger school, 52% of the votes marked the option “I liked it extremely” and 25% the option “I liked it moderately”. As for acceptance and adherence rates, they were 90.64% and 86.44%. By school, 96.17% and 83.13% (smallest) and 88.70% and 83.65% (larger). Seven of the thirteen menus served (larger size) had low adherence, and eight had low student acceptance. Four of the sixteen menus in the smaller school had low adherence, and all were well accepted. Snacks had significantly higher averages in the adherence and acceptability index concerning meals, and both indices were adequate as recommended.
Junta Nacional de Auxilio Escolar y Becas—JUNAEB (2017) Chile [91]	Evaluate the level of satisfaction of the users of the service provided by the school feeding program (SFP) through the application of a questionnaire to students belonging to the second cycle of primary education level (5th to 8th year) and middle school students (1st to 4th year) from all regions and provider companies in the country	SS: *n* = not informed TS: Middle and high school P: Students (*n* = 34.434)	Questionnaire (1 to 7 pts) Satisfaction: ≥5 pts Dissatisfaction: <5 pts Points for each variable: simple average of the scores for breakfast, salad, main course, and dessert (total sums/by 4) Satisfaction indicator *n*° of students with SFP who evaluate their level of satisfaction with the SFP service above 75% in year/total *nº* of students with SFP who answer the Service Quality Assessment Survey in year × 100	Satisfaction with meals (dimension *nº* 1) Acceptability (smell, taste, appearance, temperature, freshness, cooking) Variety (alternating) Quantity (portion size)	Overall satisfaction with the food received (dimension #1) was only 54% and 52% for the second primary and medium cycles, respectively. As for the sub-dimensions, for acceptability (52.4% and 51.9%), variety (61.6% and 55.1%), and quantity (49.9% and 49.3%). Regarding the variables that make up acceptability, the second basic cycle showed greater satisfaction for cooking (67.4%) and less for taste (44.1%). The average cycle is higher for temperature and cooking (70.6%) and lower for taste (39.1%).
Rocha et al. (2018) Brazil [92]	To analyze the implementation of the National School Feeding Program as a food and nutrition security policy in public schools	SS: *n* = 17 TS: Elementary school P: Students (*n* = 268)	Questionnaire Semi-structured questions (*n* = 5) (“no”, ≤3x/wk, ≥4x/wk); (very good/good, fair/poor); (yes, no); (yes, no); (never, sometimes, always, respectively)	Adherence, perception, satisfaction, importance of school meals, and the habit of taking snacks from home Adherence “satisfactory” = consumption ≥ 4x/week” Perception “satisfactory” = students considered the food as “very good” or “good”	Regarding the children’s perception of school meals, there was low adherence by 63.9% of the students. The amount served was satisfactory for 91.1% of the students, and 86.2% considered it important. As for the perception, among those who consumed it more frequently, 79.4% considered it “very good” or “good”. About taking snacks from home, 15.3% reported “always” and 64.2% “sometimes”.
Souza et al. (2018) Brazil [93]	To investigate the content of accession, acceptance, and rejection of school meals in three public schools of Atalaia do Norte, AM	SS: *n* = 3 TS: Preschool, elementary, middle, and high school P: Students (*n* = approximately 300)	5-point hedonic facial scale (6 to 10 years) or 5-point hedonic verbal scale (11 to 15 years) (“hated” to “loved’) % of rejection = weight of the rejected meal (leftovers on the plates)/weight of the distributed meal × 100 Acceptance rate = 100 − % of rejection AI = *nº* of students who consumed the meal/*nº* of students present at school × 100	Acceptance “Accepted” = ≥85% (Adding “liked” and “loved”) Method of leftover ingestion (assessment of leftovers) Adherence index	It was observed that all foods were accepted by the Escola Estadual Pio Veiga students, with the biscuit with dairy compound and the biscuit with chocolate milk presenting the highest acceptance rates. Meatballs with noodles had an acceptance rate of 67.9%. As for the adherence rate, all meals had medium to high adherence rates. Students reported a lack of seasoning in meals and an unpleasant taste.
Daniel and Moreira (2018) Brazil [94]	To know the adherence, frequency, acceptability, and quality of school feeding	SS: *n* = 4 TS: Elementary school P: Students (*n* = 271)	5-point hedonic mixed facial scale (“hated” to “loved’) Questionnaire Question (*n* = 1) (“yes” or “no”) Questionnaire Questions: objective (*n* = 2) (1 x/wk to 5 x/wk) and subjective (*n* = 1) (about the reason not to consume)	Acceptability Acceptance rate Accepted” = ≥85% (Adding “liked” and “loved”) Adherence index High (>70%), medium (50 to 70%), low (30 to 50%), and very low (<30%) Weekly attendance index	As for the adherence rate, it was 90.78%, which is considered high. The main reason for not consuming school meals was “I bring my own lunch” (35%). Regarding the frequency of weekly consumption, only 57% consume daily. As for acceptance, adding up those who mentioned liking and adoring, an index of 97% was obtained, considering the accepted food.
Kwon et al. (2018) South Korea [5]	To investigate the effect of satisfaction with the school meal program on students’ school happiness	SS: *n* = 91 TS: Elementary, middle, and high school P: Students (*n* = 2.336)	5-point Likert scale “strongly disagree” (1) to “strongly agree” (5)	Satisfaction School meal quality: texture diversity, food diversity, food appearance, nutritional balance, serving temperature, flavor, ingredient quality, seasonal menu, and serving quantity “Satisfactory” ≥ 4 pts	The average satisfaction score for school meal quality was 3.85, below 4 points corresponding to “satisfactory”. For each school level, we obtained: 4.17 (elementary school), 3.64 (middle school), and 3.49 (high school). Multiple regression analysis used to determine how school meal quality affects students’ happiness levels revealed no significant influence on students’ overall happiness levels.
Beintema et al. (2018) Colombia [95]	To evaluate the sensory acceptability of two biofortified beans against local beans at schools affiliated with the school feeding program in two departments in southwest Colombia	SS: *n* = not informed TS: Middle school P: Students (*n* = 174)	Questionnaire Questions (*n* = 2) (“liked”, “indifferent”, or “disliked”) and (“daily”, “weekly”, “monthly”, or “rarely/never”) 5-point Likert scale (with facial icons) (“dislike much” = 1 to “like very much” = 5)	General liking and frequency of consumption Sensory acceptability Color, size, smell, taste, texture, and overall mean	Regarding the general taste of local beans, 94.2% said they liked it, and 2.3% did not; 77.6% consume it weekly, and 2.4% rarely or never. As for the general taste, the beans averaged 3.81 pts. For the municipalities of Piendamó and Caicedonia, the median hedonic scores by sensory attribute of the local bean were color and texture (both 3; 4), size, smell, and taste (both 4), respectively. For all students from the two municipalities, the average score was 4 pts in all attributes. Thus, the general acceptability was considered good.
Bartolazze and Cazal (2019) Brazil [7]	To evaluate the nutritional composing adequacy of menus for NSFP and the provisions’ acceptability offered at a municipal school in São José do Calçado, ES	SS: *n* = 1 TS: Elementary school P: Students (*n* = 63)	5-point hedonic facial scale (“hated” to “loved’)	Acceptability Acceptance rate Accepted” = ≥85% (adding “liked” and “loved”)	The acceptability index of the meals was 75%, presenting an index lower than the parameter established by Brazilian legislation.
Mensah and Appietu (2019) Ghana [18]	To examine the determinants of dining hall meal satisfaction and the effect of overall satisfaction on the patronage of sources of meals among senior high school boarders in Ghana	SS: *n* = 2 TS: High school P: Students (*n* = 400)	4-point Likert scale (“very satisfied” = 4 to “very dissatisfied” = 1) Questionnaire Questions (*n* = 3) (“breakfast”, “lunch”, or “supper”/”once a day”, “two times a day”, or “three times a day”, respectively) 5-point Likert scale (“very satisfied” to “very dissatisfied”)	Satisfaction Presentation, variety, tastiness, temperature, freshness, quality, the quantity of food per meal, and overall satisfaction Eating patterns/preferences of students/dining profile of students (mealtime preference, frequency of eating dining hall food, and overall food satisfaction)	The total average satisfaction with food was 2.72. The attribute “presentation of food” presented the highest average (2.93, 76.2% satisfied), and the “quantity of food per meal” was the lowest (2.37, 45.5%). As for the student’s preferences, the majority (40.2%) preferred dinner instead of having breakfast (31.8%) or lunch (28.1%) in the cafeteria. Regarding attendance, only 38.5% of the students indicated that they attended all three meals served in the cafeterias. As for general satisfaction with food, 41.4% were satisfied, 27.7% were dissatisfied, and 30,9% were neither satisfied nor dissatisfied. Food taste, quality, and variety of meals were the key predictors of overall meal satisfaction. Overall, food service satisfaction was related to the patronage of sources of meals.
Souza et al. (2019) Brazil [96]	To evaluate the influence of an intervention on the nutritional and sensory quality of the menus and the food waste of a children’s educational center	SS: *n* = 1 TS: Preschool P: Students (*n* = 45)	Waste ingestion per child (kg) = weight of the total waste/*nº* of children Percent waste ingestion (%WI) = weight of the plate waste/weight of the distributed meal × 100 Clean leftovers per child (kg) = weight of the clean leftovers total/*nº* of children Percent clean leftovers (%CL) = weight of the clean leftovers/weight of the produced meal × 100	Waste ingestion (WI) %WI = ≤ 10% Clean leftovers (CL) %CL ≤ 3% or 7 to 25 g/child	As for waste ingestion, the average total amount was 2.16 kg, average WI/child of 69.02 g, and %WI of 29.68%, exceeding the tolerable limit of 10%. Regarding clean leftovers, the total average amount was 5.03 kg, the average CL/child was 161.47 g, and %CL was 39.55%, above the acceptable limit of 25 g/child.
Niño-Bautista et al. (2019) Colombia [97]	To determine the prevalence of perception of satisfaction of the beneficiaries of the school feeding program in Bucaramanga, Colombia, and its associated factors	SS: *n* = 18 TS: Elementary, middle, and high school P: Students (*n* = 401)	Sensory component Questionnaire Questions (*n* = 3) (“yes” or “no”) Scores: 1 point for each affirmative answer (positive aspects) 1 to 27 pts Group 1: (9 to 17 y) Group 2: (5 to 8 y) Both groups, if they received industrialized or prepared on-site food	Perception of satisfaction Sensory component (taste, color, and smell) Perception of satisfaction according to the type of food received	Students in group 1 showed greater satisfaction in the aspects evaluated. The industrialized and locally prepared meals obtained the following satisfaction percentages for flavor: 96.77 and 96.55% (G1), 82.45 and 80.52% (G2); smell: 98.39 and 96.55% (G1), 80.65 and 87.90% (G2); color: 80.65 and 84.48% (G1), 52.42 and 59.24% (G2). When comparing satisfaction scores and type of meal by age group, the lowest score (4.14) was for group 2 in the industrialized meal and the highest for group 1, who received a meal prepared on site.
Lee (2019) South Korea [98]	To compare student consumption of school meals by school level, to identify the influencing factors of school meal consumption, and to assess improvement needs of school food service among students	SS: *n* = 58 TS: Elementary, middle, and high school P: Students (*n* = 1.441)	5-point scale (“eat all served” = 1 to “eat none” = 5) and (“very small” = 1 to “very large” = 5) 5-point Likert scale (“strongly disagree” = 1 to “strongly agree”) Less = suggested reasons (*n* = 11) or More = suggested reasons (*n* = 8) 5-point scale (“very dissatisfied” = 1 to “very satisfied” = 5) 5-point Likert scale (“very unnecessary” = 1 to “very necessary” = 5) Suggested reasons (*n* = 16) no total answered through	Consumption and perception of the portion served Reasons for eating: Less (for those who had less than half the portion served) More (for those who had half, or more than half, the portion served) Satisfaction (sanitation, temperature, presentation, taste, variety of menus, portion size, and reflecting student opinions) Needless to improve school food service to increase consumption	A total of 76.1% consumed almost all or all of the meals served. Approximately 58% of students perceived the school lunch portion size as adequate. The main reason for consuming half or more than half of the portion was “because the food tastes good” (3.65), and consuming less than half was “because the food is not tasty” (3.76). The mean satisfaction scores for all students evaluated were sanitation (3.80), temperature (3.72), presentation (3.64), taste (3.63), variety of menus (3.56), portion size (3.43), and reflecting student opinions (3.31). All independent variables were significant predictors of meal consumption. Students with higher scores for eating behavior, satisfaction with food service, environmental protection, and more positive behavior scores and attitudes toward school meals consumed significantly more meals. Regarding improving the school food service, “serving food that students prefer” (4.18 points) was the most prevalent.
USDA (2019) USA [99]	To assess the student participation, student and parent satisfaction, plate waste, and students’ dietary intakes from school meal programs (Volume 4)	SS: *n* = 1.200 TS: Elementary, middle, and high school P: Students (*n* = 1.733) for views on foods served for lunch. Overall satisfaction with lunch (*n* = 1.215) and breakfast (*n* = 464) Parents (*n* = 1.850) for healthiness, (*n* = 1.504) for satisfaction, the child likes school lunches and somewhat or very dissatisfied, (*n* = 272) for reasons for dissatisfaction	Interview Satisfaction with lunch (questions *n* = 13) Saltiness: (“about right”, “not salty enough”, “too salty”, or “missing”) Amount of food (portions): (“about right”, “too little”, “too much”, or “missing”) For the rest (“always”, “often”, “sometimes”, “never”, or “missing”) Students in grades 4–12 grades (“Do you like it”, “think it is only okay”, or “not like it?”) and Students in grades 1–3 (facial expressions and 3 response options: (“likes school lunch/breakfast”, “schools lunch/breakfast Is only okay”, or “does not like school lunch/breakfast”) Healthiness (“very healthy”, “somewhat healthy”, “not healthy”, “It depends”, or “don’t know”) If child likes (“strongly agree”, “agree somewhat”, “disagree somewhat”, “strongly disagree”, or “missing”) Satisfaction (“very satisfied”, “somewhat satisfied”, “somewhat dissatisfied”, “very dissatisfied”, “don’t know”) (“somewhat” or “very dissatisfied”) Reasons (*n* = 12)	Satisfaction Students’ views on foods served for lunch (availability of foods they like, number of choices offered, look and smell of the food) General satisfaction with school lunches and breakfast Parents’ views on school lunches (healthiness, if the child likes school lunches and satisfaction) The same questions for school breakfast Parents’ reasons for dissatisfaction with school lunches	The results showed the student’s opinions about the foods served at lunch. Most responded “sometimes” for “lunch menu includes foods they like”, “like the way the food looks”, “like the smell of the food looks”, “like the vegetables in the serving line”, “vegetables in serving line look good”, “enough food choices”, for “like the taste of the food” and “likes the whole grain foods available”. Most students responded “always” for “serving line has milk they like” (60%), “like the fruits in the serving line” (37%), and “fruits in serving line look good” (42.7%). For “saltiness of food served” and “amount of food (portions)” 79.4% and 72.7% responded “about right”, respectively. Regarding the general satisfaction with school lunches and breakfast, 36% and 56.5% of students liked it, 52% and 37.9% reported that it was only okay, and 12% and 5.9% reported they did not like it, respectively. In the parents’ view of school lunches for “healthiness”, 62.8% considered “somewhat healthy”. About “child likes”, 50.9% reported that they “agree somewhat”, and about “satisfaction”, 52.1% said they were “somewhat satisfied”. A total of 19.4% were “somewhat” or “very dissatisfied”, and the main reason for dissatisfaction was “poor quality/taste”.
Assan et al. (2020) India [100]	To examine the impact of an internationally funded Indian foundation’s mid-day meal (MDM) school feeding program on educational access, performance, participation, and well-being of the beneficiaries	SS: *n* = 62 TS: Elementary, middle P: Students (*n* = 1.338), teachers (175)	4-point hedonic scale (“poor”, “good”, “very good”, “excellent”) 3-point hedonic scale (“not satisfied”, “satisfied”, “very satisfied)	Satisfaction level Quality (general, taste, flavor, variety, presentation) Quantity of meal/potion served (general, freedom to ask more, quantity per week)	Adding the percentages of “good”, “very good” and “excellent”, students and teachers rated the general quality (97 and 99%), flavor (96 and 99%), and taste (95 and 97%), respectively. As for the presentation of the menu, only 1% and 2% were dissatisfied. Already 9% of both were dissatisfied with the variety of the menu. Regarding quality improvement, the majority, 27% of students and 56% of teachers, suggested options to increase menu variation. As for the amount of food, 2% of the students and 5% of the teachers did not find an adequate amount. Already weekly, 1% and 6%. As for the “freedom to ask for more”, 2% of students and 1% of teachers were dissatisfied.
Joyce et al. (2020) USA [101]	To compare the acceptability and feasibility of best practice (BPSL, optimizing DQ) with typical school lunches (TSL, meeting minimum NSLP standards) served separately and concurrently	SS: Local school districts (*n* = 4) TS: Elementary school P: Students (*n* = 36)	5-point Likert scale (with smiley faces) (“very bad” = 1 to “very good” = 5) and Any comments? Average total plate waste value and photos Hunger scale (“How hungry are you?”) 5-point Likert scale (stuffed = 1, full = 2, comfortable = 3, hungry = 4, ravenous = 5)	Acceptability Taste test (appearance, smell, taste, and desire to serve at school) Plate waste Change in hunger (level of satiety before and after consumption) CH: pre-meal hunger subtracted from post-meal hunger	The typical school lunch (TSL) had the average scores: taste (4.6), smell (4.3), appearance (4.4), and serving at school (4.5). The average total meal waste was 47.8%, with the highest waste for milk (68.8%) and the lowest for proteins (28.3%).
Peres et al. (2020) Brazil [102]	To evaluate, through sensory analysis, the acceptability of goat’s milk over cow’s milk in public schools in the municipality of Bambuí, MG	SS: *n* = 2 TS: Elementary, high P: Students (*n* = 330)	5-point hedonic scale (“liked it a lot = 5 to “disliked it very much" = 1)	Acceptability Acceptance test	Cow’s milk (C) had an average score of 3.77 on the acceptance test. When checking the preference for school, an average of 3.88 (school 1) and 3.71 (school 2) was observed.
Guimarães (2020) Brazil [103]	To assess the nutritional quality and acceptability of school food cards in a municipality in Bahia	SS: *n* = 5 TS: Elementary, middle P: Students (*n* = 445)	5-point hedonic facial scale (1st to 3rd grade) or 5-point hedonic mixed facial scale (4th and 5th grade) 5-point hedonic verbal scale (6th to 8th grade) (“hated” to “loved’)	Acceptance	There was good acceptance of school meals among students. When asked about the foods they would like included on the menus, of the 445 students who responded, they included fruit salad (41.3%), hot dogs (39.2%), and soda (28.12%). The least accepted menu was coconut hominy (3.91–61.6%), and the most accepted was carrot cake with yogurt (4.62–92.4%).
Donadini et al. (2021) Italy [104]	To explore meal liking and to understand mechanisms behind meal acceptance	SS: *n* = 1 TS: Preschool P: Students (*n* = 60)	5-point nongender horizontally oriented facial scale Verbally articulated the to dish liking (“super-bad”, “bad”, “so and so”, "good”, “super-good”), indicated the facial icon, and inserted a scoring card into 1 of 5 boxes lined up in front of him/her. Referring to the score of (1 = “dislike much” to 5 = “like very much”)	Acceptance Liking (overall) and reported familiarity by parents (about the served dishes)	As for the taste of the dishes, the average was 4.1. Among the first course, second course, side dish, and dessert or fruit, the dishes with the highest and lowest scores were: risotto with saffron (4.9) and pureed vegetable soup (4.4); braised loin (4.3) and oven-baked breaded plaice (3.7); raw carrots (4.1) and raw fennels (2.6); delicious golden apple (4.7) and red-orange juice (3.4), respectively. The familiarity reported by the parents showed that cheese and tomato pizza and risotto with saffron (4.7) were familiar to the children, and raw fennels (2.7) were the least familiar. Taste scores correlated with food neophobia, familiarity, sweetness, odor, and flavor intensity. The mother’s taste was most strongly associated with the child’s taste.
Araya and Castillo-Montes (2021) Chile [105]	To determine the degree of acceptability of prepared lunches that are given to students by the SFP and its association with economic losses	SS: *n* = 5 TS: Elementary, middle, and high school P: Students (*n* = 528) Food handlers and teachers in charge of the SFP (*n* = 5)	3 instruments: (1) 9-point hedonic scale (1 = “dislike extremely” to 9 = “like extremely“) The results were grouped into four categories of preferences (A, B, C, and, D) where category A presents the highest preference and the following ones in descending order up to D. and The students were asked about their perceptions of the organoleptic characteristics: Smell, flavor, appearance, and consistency (good, regular, or bad) to Salt content (with salt−without salt), cooking (adequate−inadequate). temperature (hot–cold) and quantity of the portion (sufficient−insufficient) (2) Sorting test: sensory estimation analysis to determine the difference in magnitude according to the degree of preference of the six preparations, based on the criteria of food handlers and teachers in charge of the PAE, who ordered the preparations according to the degree of preference they observed in the students. The ordering test scale was scores of 1 to 6 (1 = highest preference and 6 = least) (3) The percentage of intake was determined in quartiles (25, 50, 75, and 100%) about the amount of main course not eaten (salads and desserts were not considered)	Acceptability	The thinly sliced beef with noodles was found to have the highest preference and the lowest-ranked meals were beans with noodles and fish cake with mashed potatoes (p < 0.05). Preparations with lower acceptability represented 82.2% of economic losses and were mainly beans. The results confirm a low intake of fish and vegetables, that taste characteristics due to low salt content affect preferences, and that there was a direct relationship between intake and economic loss.
Donadini et al. (2022) Italy [106]	To explore pre-schoolers liking of meals and of individual dishes served in a meal by tasting actual foods in the natural setting of the school canteen	SS: *n* = 1 TS: Preschool P: Students (*n* = 127) Teachers (*n* = 3)	5-point nongender horizontally oriented facial scale (super-bad to super-good) a one-on-one interview with the teachers The child declared loudly how much s/he had liked the meal using the scale descriptors and indicated to what facial icon the meal belonged by pointing to one of the five boxes lined up in front of him/her Visual estimation (by elected teachers) 7-point scale (all, one mouthful eaten, ¾, ½, ¼, one mouthful left, none) Maximum score = no food remained on the plate Intermediate score = ¾, ½, ¼ of the food, or just a bit of the food remained Minimum score = the plate was left untouched Percentage of the uneaten food for each child and each dish: estimated to the nearest 10% increment (considering the appearance of full servings) The scores on the scale were converted to weight estimates of plate waste for all servings based on initial weights: multiplying the initial weight of each serving by the percentage value corresponding to the score given on the scale (7 = 100%; 6 = 90%; 5 = 75%; 4 = 50%; 3 = 25%; 2 = 10%; 1 = 0%)	Overall liking Plate waste The leftovers of each child and vegetable and fish items were recorded, and the relation between the uneaten amount, liking of and familiarity to these items was studied	Liking varied (p < 0.001) across meals and individual dishes. The most appreciated meals included: solid starch-based dishes with meat sauce and cheese, lean poultry meat, roast potatoes, fruit yogurt, fresh seasonal fruit, or fruit ice cream. Least liked meals included vegetable soup (with pasta or rice), pasta with zucchini or legumes, seafood, or cheese. Fish and cheese were moderately liked, and vegetables were liked the least. The first and second courses contributed most to overall meal acceptability. Liking of side dishes was uncorrelated to individual meal liking in most children. Children liking and reported familiarity by parents successfully predicted the amount of food eaten. Girls were more familiar with vegetable dishes and likelier to like and consume these dishes than boys. Children’s sex did not affect fish consumption.
Pinto (2022) Brazil [107]	To evaluate the effectiveness of a multicomponent intervention in adherence and acceptability to school meals (A)	SS: *n* = 3 TS: Elementary and middle school P: Students (sample estimates: *n* = 90 for acceptability, and *n* = 396 for adherence)	One school in each group: Control group: no intervention Group 1: environmental modifications and addition of a self-service system Group 2: environmental modifications, the addition of a self-service system, and the inclusion of new dishes in the menu 5-point mixed facial hedonic scale (4th to 5th grade) 5-point verbal hedonic scale (6th to 9th grade) (“hated” to “loved”) Objective question (*n* = 1) “Do you eat the lunch offered by the school?” with six answer options (“never or almost never”; “1x/week” to “4x/week”; “every day”)	Acceptability index Average acceptability: Positive: ≥4 Negative: <4 Adherence ≥3 times/week	There was a significant increase in the acceptability index in the groups of preparations based on meat and sweets among participants in intervention school 1 and intervention 2, compared to the control school over time. The increase in fruit acceptability was greater among participants in intervention school 2 when compared to those in intervention school 1. Reduction in the acceptability of bean-based preparations was observed between intervention school 2 and intervention 1 and control schools. An increase in adherence to school meals was observed in the intervention school 1 vs. control school and a greater increase in intervention school 2 vs. control school.
Pinto (2022) Brazil [107]	To identify the factors that could contribute to the low adherence and acceptability of the food offered at school (B)	SS: *n* = 3 TS: Elementary and middle school P: Students and cooks (*n* = 5 to 8 people in each group)	Two focus groups (with students from the one school): The students who report the consumption and non-consumption of the meals offered Subjective questions (*n* = 7, each) One focal group (with cooks from the three schools): Subjective questions (*n* = 6) Regarding self-knowledge about the role of cooks in the school unit; perception of adherence and acceptability of food by students; motivation for non-consumption of preparations by students; way of preparing and distributing meals and what actions could be taken to make food more attractive to students	Adherence and acceptability	Based on the perceptions about the reasons for non-adherence to school meals reported in focus groups carried out with lunch ladies and schoolchildren, the standardization of gastronomic techniques was possible, leading to changes in the dishes that were part of the menus of the schools and also the creation of new ones. These new menus were offered for group 2 of the previous study.

**Table 2:** Distribution of sensory evaluation methods and acceptance of school menus of school feeding programs among the included studies.

Sensory Evaluation and Acceptance Methods Performed	Evaluated Attributes	Number of Studies *	%
Hedonic/Likert scale (2 to 9 points)	Acceptability Acceptance Satisfaction Preference	62	69.66
Mathematical formulas/visual estimate (evaluation of consumption and leftovers)	Leftover food	36	40.45
Qualitative methodology of collective subject discourse (CSD)	Acceptance	1	1.12
Questionnaire/Interviews (objective and subjective questions)	Acceptability Acceptance Satisfaction Preference Adherence Leftover food	40	44.94

Note: The total sum exceeded one hundred percent (100%) as the same study used two or more methods. * Total number of studies (*n* = 89).

**Table 3:** Distribution of sensory evaluation methods and acceptance of school menus of school feeding programs among the included studies. Distribution of the studies by country and sensory evaluation and acceptance methods of school menus in SFPs.

Country	Number of Studies	Main Method of Sensory Evaluation and Acceptance	Number of Studies that Used the Method	%
Brazil	42	Questionnaire and interview (Objective and subjective questions)	26	61.90
South Korea	13	Hedonic/Likert scale (4, 5, and 6 points)	13	100
United States	12	Hedonic/Likert scale (3, 5, and 9 points)	9	75
Italy	8	Hedonic scale (4, 5, and 7 points)	6	75
Colombia	2	Questionnaire (objective questions)	2	100
India	2	Hedonic/Likert scale (2, 3, and 4 points)	2	100
Chile	3	Mathematical formulas (evaluation of consumption and leftovers)	2	66.67
Ghana	1	Likert scale (4 points) and Questionnaire (objective questions)	1	100
Philippines	1	Hedonic scale (7 points)	1	100
Spain	1	Mathematical formulas and visual estimate (evaluation of consumption and leftovers)	1	100
Paraguay	1	Hedonic scale (5 points) and Mathematical formulas (evaluation of consumption and leftovers)	1	100
Finland	1	Likert scale (7 points) and Questionnaire (subjective questions)	1	100
Georgia	1	Visual estimate (evaluation of consumption and leftovers) and interview (objective and subjective questions)	1	100

## Data Availability

Not applicable.

## Supplementary Materials

The following supporting information can be downloaded at: https://www.mdpi.com/article/10.3390/ijerph20032242/s1, Table S1. Full-text articles excluded with reasons; Table S2. Database and terms used to search references on the methods of sensory evaluation and acceptance of school menus from school feeding programs around the world; Table S3. Quality criteria of the selected studies for the systematic review of the sensory evaluation methods and acceptance of school menus from school feeding programs around the world.
