# Supplementary material for: Acceptability of School Menus: A Systematic Review of Assessment Methods
Source: Int J Environ Res Public Health. 2023 Jan 27;20(3):2242. doi: 10.3390/ijerph20032242 (PMC9914958; doi:10.3390/ijerph20032242)
Supplement: Supplementary file 1 [file ijerph-20-02242-s001.zip › ijerph-2105100-supplementary.pdf]

Supplementary data

Table S1. Full-text articles excluded, with reasons.

Author, year	Exclusion Motif
Elramlil and Elbakoush (2019)	2
Gewa et al. (2005)	1
Puche (2020)	3
Homann et al. (2017)	3
Kinyuru et al. (2021)	3
Osei et al. (2008)	3
Anitha et al. (2019)	3
Vale et al. (2021)	2
Davidson (1979)	2
Damsgaard et al. (2012)	2
Sampat and Tepper (2019)	2
Ramos et al. (2020)	2
Nguyen (2018)	2
Favuzzi et al. (2020)	2
Bustamente et al. (2018)	2
Howe and Vaden (1980)	2
Miller (1996)	2
Rodríguez-Tadeo et al. (2014)	2
Marlette et al. (2005)	2
Sepúlveda et al. (2011)	3
Latorres et al.(2016)	3
Donadini et al. (2013)	2
Olivares et al. (1990)	3
Fritts et al. (2018)	3
Cohn et al. (2013)	2
Hamdi et al. (2020)	3
Kjosen and Moore (2014)	3
Ali and Akbar (2015)	2
Longo-Silva et al. (2013)	2
Stracener and Nettles (1999)	2
Issa et al. (2014)	2
Colombo et al. (2020)	3
Baxter et al. (1998)	2
Meyer (2005)	2
Meyer and Conklin (1998)	2
Barbosa et al. (2006)	2
Pedraza et al.(2007)	2
Sanches (2002)	2
Asperin et al. (2010)	2
Sturion et al. (2005)	2
Vieira (2008)	1
Ferreira (2008)	2
Álvares et al. (2008)	2
Oliveira and Vassimon (2012)	1
Mota et al. (2013)	2
Rossi et al. (2014)	2
Baxter et al. (2000)	3
Jeong et al. (2013)	2
Park and Choe (2015)	2
Lowe et al. (2004)	3
Baxter et al. (1999)	3
Noble et al. (2000)	2
Gutsch (1977)	2
Rieley (1986)	3

Liang and Zhang (2009)	2
Marples and Spillman (1995)	2
Meyer (2000)	1
Yi (2012)	2
Cunha et al. (2014)	3
Hernández et al. (2008)	2
Leal et al. (2010)	2
Condon et al. (2009)	2
Schwartz (2007)	2
Ratcliffe et al. (2011)	2
Jaramillo et al. (2006)	2
Yoder et al. (2014)	2
Bruselius-Jensen (2014)	2
Lülfs-Baden et al. (2008)	2
Parmer et al. (2009)	3
Endres et al. (2003)	2
Yoon and Kim (2012)	2
Kim et al. (2011)	2
Choi and Han (2009)	2
Ochsenhofer et al. (2006)	2
Bartrina et al. (2004)	4
Kwon et al. (2005)	2
Kim et al. (2007)	2
Kim et al. (2000)	2
Haas et al. (2014)	2
Christmann (2011)	1
Silva et al. (2010)	1
Sturion et al. (2004)	1
Caporale et al. (2003)	1
Ferro (2003)	1
Silva (2018)	1
Donadini et al. (2009)	1
Santos et al. (2010)	1
Kim et al. (2002)	2
Kim (2013)	2
Pinto (2019)	5
Helland et al. (2021)	5
Rossi et al. (2021)	2

Legend - Exclusion criteria: 1) Comments, reviews, letters, abstracts, conferences, undergraduate papers, clinical and review studies, case reports, and books (n = 13); 2) Studies that do not focus on the evaluation of acceptance of school menus from School Feeding Programs around the world (n = 61); 3) Studies that analyzed the acceptance of preparations that have not yet been included in the menus of the SFP (n = 18); 4) Studies in private schools, unrelated to government-subsidized SFP (n = 1), 5) incomplete studies (n = 2) and (6) studies that evaluated the acceptance of menus offered in Universities (n = 0).

Table S2. Database and terms used to search references on the methods of sensory evaluation and acceptance of school menus from School Feeding Programs around the world.

Database	Search (Jan 11th, 2023)
PubMed 304	((((((((((((((((((((("school feeding" [Title/Abstract]) OR ("school feeding program"[Title/Abstract])) OR ("school feeding programs" [Title/Abstract])) OR ("school feeding programmes"[Title/Abstract])) OR ("school food service" [Title/Abstract])) OR ("school food services" [Title/Abstract])) OR ("national school food program" [Title/Abstract])) OR ("school meal"[Title/Abstract])) OR ("school meals" [Title/Abstract])) OR ("school menu" [Title/Abstract])) OR ("school menus" [Title/Abstract])) OR ("school lunch" [Title/Abstract])) OR ("school lunches" [Title/Abstract])) OR ("school canteens" [Title/Abstract])) AND (acceptance [Title/Abstract])) OR (acceptability [Title/Abstract])) OR (acceptation [Title/Abstract])) OR (adhesion [Title/Abstract])) OR (adherence [Title/Abstract])) OR (satisfaction [Title/Abstract])) AND ("sensory analysis"[Title/Abstract]))
Lilacs 12	("school feeding") OR ("school feeding program") OR ("school feeding programs") OR ("school feeding programmes") OR ("school food service") OR ("school food services") OR ("national school food program") OR ("school meal") OR ("school meals") OR ("school menu") OR ("school menus") OR ("school lunch") OR ("school lunches") OR ("school canteens") AND (acceptance) OR (acceptability) OR (acceptation) OR (adhesion) OR (adherence) OR (satisfaction) AND ("sensory analysis") OR ("sensory attributes")
Web of Science 667	TS=(“school feeding”) OR TS=(“school feeding program”) AND TS=(“meal acceptance”) OR TS=(“meal acceptability”) OR TS=(“meal acceptation”) AND TS=(“sensory analysis”)
Scopus 117	(ALL (“school feeding”) OR ALL (“school feeding program”) OR ALL (“school feeding programmes”) OR ALL (“school food service”) OR ALL (“school food services”) OR ALL (“national school food program”) OR ALL (“school meal”) OR ALL (“school meals”) OR ALL (“school menu”) OR ALL (“school menus”) OR ALL (“school lunch”) OR ALL (“school lunches”) OR ALL (“school canteens”) AND ALL (“acceptance”) OR ALL (“acceptability”) OR ALL (“acceptation”) OR ALL (“adhesion”) OR ALL (“ adherence”) OR ALL (“satisfaction”) AND ALL (“sensory analysis”) OR ALL (“sensory attributes”))
Embase 474	(('school feeding' OR 'school feeding program' OR 'school feeding programs' OR 'school feeding programmes' OR 'school food service' OR 'school food services' OR 'national school food program' OR 'school meal'/exp OR 'school meal' OR 'school meals' OR 'school

	menu' OR 'school menus' OR 'school lunch' OR 'school lunches' OR 'school canteens') AND ('acceptance'/exp OR acceptance) OR 'acceptability'/exp OR acceptability OR acceptation OR 'adhesion'/exp OR adhesion OR 'adherence'/exp OR adherence OR 'satisfaction'/exp OR satisfaction) AND ('sensory analysis'/exp OR 'sensory analysis')
Proquest 445	ab("school feeding" OR "school feeding program" OR "school feeding programs" OR "school feeding programmes" OR "school food service" OR "school food services" OR "national school food program" OR "school meal" OR "school meals" OR "school menu" OR "school menus" OR "school lunch" OR "school lunches" OR "school canteens") AND ab("acceptance" OR "acceptability" OR "acceptation" OR "adhesion" OR "adherence" OR "satisfaction" OR "sensory analysis" OR "sensory attributes")
Google Scholar<br>400 more relevant<br>9.350 results	("school feeding" OR "school feeding program") AND ("acceptance" OR "satisfaction" OR "sensory analysis")

Table S3. Quality criteria of the selected studies for the systematic review of the sensory evaluation methods and acceptance of school menus from School Feeding Programs around the world.

Reference	1. Were the methods of evaluation of acceptance of menus characterized?	2. Were the evaluated menus and/or preparations specified?	3. Was the evaluation carried out in schools participating in School Feeding Programs?	4. Was the study design adequate?	5. Was the sample of participants selected for the analysis representative and/or randomly determined?	6. Was the statistical analysis adequate to the objective of the study?	7. Did the results answer the main question?	Percentage of positive responses (yes) for each study that attained the quality criteria
Head et al. (1977)	Y	Y	Y	Y	N	Y	Y	85.71%
Devan et al. (1988)	Y	Y	Y	Y	N	N	Y	71.42%
Stalls (1997)	Y	Y	Y	Y	N	Y	Y	85.71%
Kim and Kim (1997)	Y	Y	Y	Y	Y	Y	Y	100%
Baxter et al. (2000)	Y	Y	Y	Y	Y	Y	Y	100%
Brandão (2000)	Y	Y	Y	Y	Y	Y	Y	100%
Sturion (2002)	Y	Y	Y	Y	N	Y	Y	85.71%
Pagliarini et al. (2003)	Y	Y	Y	Y	N	Y	Y	85.71%
Hong and Chang (2003)	Y	Y	Y	Y	Y	Y	Y	100%
Flávio et al. (2004)	Y	Y	Y	Y	N	Y	Y	85.71%
Martins et al. (2004)	Y	Y	Y	Y	N	N	Y	71.42%
Pagliarini et al. (2005)	Y	Y	Y	Y	N	Y	Y	85.71%
Lee and Lyu (2005)	Y	Y	Y	Y	N	Y	Y	85.71%
Yoon et al. (2005)	Y	Y	Y	Y	N	Y	Y	85.71%
Jang and Kim (2005)	Y	Y	Y	Y	N	Y	Y	85.71%
Lee and Jang (2005)	Y	Y	Y	Y	Y	Y	Y	100%
Stroebele et al. (2006)	Y	Y	Y	Y	N	Y	Y	85.71%
Pecorari (2006)	Y	Y	Y	Y	Y	Y	Y	100%

Flávio (2006)	Y	Y	Y	Y	Y	Y	Y	100%
Rossi et al. (2006)	Y	Y	Y	Y	N	Y	Y	85.71%
Byun and Jung (2006)	Y	Y	Y	N	N	Y	Y	7142%
Muniz and Carvalho (2007)	Y	Y	Y	Y	N	Y	Y	85.71%
Conrado and Novello (2007)	Y	Y	Y	Y	N	Y	Y	85.71%
Danelon (2007)	Y	Y	Y	Y	Y	Y	Y	100%
Park and Jang (2008)	Y	Y	Y	Y	Y	Y	Y	100%
Santos et al. (2008)	Y	Y	Y	Y	Y	N	Y	85.71%
Danelon et al. (2008)	Y	Y	Y	Y	Y	Y	Y	100%
Abranches et al. (2009)	Y	Y	Y	Y	N	Y	Y	85.71%
Teo et al. (2009)	Y	Y	Y	Y	Y	Y	Y	100%
Bleil et al. (2009)	Y	Y	Y	Y	Y	Y	Y	100%
Caporale et al. (2009)	Y	Y	Y	Y	N	Y	Y	85.71%
Song and Moon (2010)	Y	Y	Y	Y	N	Y	Y	85.71%
Lazor et al. (2010)	Y	Y	Y	Y	N	Y	Y	85.71%
Pegolo and Da Silva (2010)	Y	Y	Y	Y	Y	Y	Y	100%
Matihara et al. (2010)	Y	Y	Y	Y	N	Y	Y	85.71%
Lee and Park (2010)	Y	Y	Y	Y	N	Y	Y	85.71%
Chu et al. (2011)	Y	Y	Y	Y	N	Y	Y	85.71%
Chesser (2013)	Y	Y	Y	Y	N	Y	Y	85.71%
Dias et al. (2013)	Y	Y	Y	Y	N	Y	Y	85.71%
Cruz et al. (2013)	Y	Y	Y	Y	Y	Y	Y	100%

Barrios et al. (2013)	Y	Y	Y	Y	N	Y	Y	85.71%
Leme et al. (2013)	Y	Y	Y	Y	N	Y	Y	85.71%
Da Silva et al. (2013)	Y	Y	Y	Y	Y	Y	Y	100%
Yang et al. (2013)	Y	Y	Y	N	N	Y	Y	71.42%
Turconi et al. (2013)	Y	Y	Y	Y	N	Y	Y	85.71%
Tadeo et al. (2014)	Y	Y	Y	Y	Y	Y	Y	100%
Angeles-Agdeppa et al. (2014)	Y	Y	Y	Y	Y	Y	Y	100%
Valeriani and Sturion (2014)	Y		Y	Y	N	Y	Y	85.71%
De Oliveira et al. (2015)	Y	Y	Y	Y	N	Y	Y	85.71%
Carlini et al. (2015)	Y	Y	Y	Y	Y	Y	Y	100%
Smith (2015) Chapter 3	Y	Y	Y	Y	Y	Y	Y	100%
Smith (2015) Chapter 4	Y	Y	Y	N	N	Y	Y	71.42%
Tuorila et al. (2015)	Y	Y	Y	Y	N	Y	Y	85.71%
Ali and Akbar (2015)	Y	Y	Y	N	Y	Y	Y	85.71%
Basaglia et al. (2015)	Y	Y	Y	Y	N	N	Y	71.42%
Ferreira et al. (2015)	Y	Y	Y	Y	N	Y	Y	85.71%
Smith et al. (2015)	Y	Y	Y	N	N	Y	Y	71.42%
Silva et al. (2016)	Y	Y	Y	Y	N	N	Y	71.42%
Balestrin et al. (2016)	Y	Y	Y	Y	N	Y	Y	85.71%
Da Silva and Barros (2016)	Y	Y	Y	Y	Y	N	Y	85.71%

Maietta and Gorgitano (2016)	Y	Y	Y	Y	Y	Y	Y	100%
Bez (2017)	Y	Y	Y	Y	N	Y	Y	85.71%
Sanabria et al. (2017)	Y	Y	Y	Y	N	N	Y	71.42%
Da Silva et al. (2017)	Y	Y	Y	Y	N	Y	Y	85.71%
Carvalho et al. (2017)	Y	Y	Y	Y	Y	Y	Y	100%
Pedraza et al. (2017)	Y	Y	Y	Y	Y	Y	Y	100%
Raphaelli et al. (2017)	Y	Y	Y	Y	Y	Y	Y	100%
Junta Nacional de Auxilio Escolar y Becas – JUNAEB (2017)	Y	Y	Y	Y	Y	Y	Y	100%
Rocha et al. (2018)	Y	Y	Y	Y	Y	Y	Y	100%
Souza et al. (2018)	Y	Y	Y	Y	N	N	Y	71.42%
Daniel and Moreira (2018)	Y	Y	Y	Y	N	N	Y	71.42%
Kwon et al. (2018)	Y	Y	Y	Y	N	Y	Y	85.71%
Beintema et al. (2018)	Y	Y	Y	Y	N	Y	Y	85.71%
Bartolazze and Cazal (2019)	Y	Y	Y	Y	N	N	Y	71.42%
Mensah and Appietu (2019)	Y	Y	Y	Y	N	Y	Y	85.71%
Souza et al. (2019)	Y	Y	Y	Y	N	Y	Y	85.71%
Niño-Bautista et al. (2019)	Y	Y	Y	Y	Y	Y	Y	100%
Lee (2019)	Y	Y	Y	Y	Y	Y	Y	100%
USDA (2019)	Y	Y	Y	Y	Y	Y	Y	100%
Assan et al. (2020)	Y	Y	Y	Y	Y	Y	Y	100%

Joyce et al. (2020)	Y	Y	Y	Y	Y	Y	Y	100%
Peres et al. (2020)	Y	Y	Y	Y	N	Y	Y	85.71%
Guimarães (2020)	Y	Y	Y	Y	Y	Y	Y	100%
Donadini et al. (2021)	Y	Y	Y	Y	N	Y	Y	85.71%
Araya and Castillo-Montes (2021)	Y	Y	Y	Y	Y	Y	Y	100%
Donadini et al. (2022)	Y	Y	Y	Y	N	Y	Y	85.71%
Pinto (2022) (A)	Y	Y	Y	Y	Y	Y	Y	100%
Pinto (2022) (B)	Y	Y	Y	Y	Y	Y	Y	100%
Txurruka et al. (2023)	Y	Y	Y	Y	Y	Y	Y	100%

Legend: Y – yes; N – no; NA – not applicable.
